# Multimodal Wearable Biosensing Meets Multidomain AI: A Pathway to Decentralized Healthcare

**DOI:** 10.1002/advs.202522900

**Published:** 2026-02-12

**Authors:** Chenshu Liu, Haolin Fan, Minwoo Kim, Tong Zhou, Pinyi Yang, Lingdi Zhao, Yiran Wang, Ziyuan Che, Chia‐Wei Liu, Bingbing Li, Yangzhi Zhu

**Affiliations:** ^1^ Autonomy Research Center for STEAHM (ARCS) California State University Northridge Northridge California United States; ^2^ Terasaki Institute for Biomedical Innovation Los Angeles California United States; ^3^ Department of Mechanical Engineering National University of Singapore Singapore Singapore; ^4^ School of Engineering and Applied Science University of Virginia Charlottesville Virginia United States; ^5^ Institute of Data Science Columbia University New York New York United States; ^6^ Fashion Management Parsons School of Design The New School New York New York United States; ^7^ Samueli School of Engineering University of California Los Angeles California United States; ^8^ Department of Chemistry and Chemical Biology Harvard University Cambridge Massachusetts United States; ^9^ School of Optometry Indiana University Bloomington Indiana United States

**Keywords:** Biosensors, digital health, flexible bioelectronics, large language model, multi‐domain AI

## Abstract

Recent advances in multimodal wearable biosensing enable continuous, noninvasive or minimally invasive monitoring of physical, physiological, and biochemical states in daily life. In parallel, multidomain AI architectures are increasingly capable of fusing heterogeneous streams, creating new opportunities for scalable, patient‐specific health analytics. Yet many sensor–AI systems remain narrow, tracking limited parameters, and often emphasize real‐time signals while underutilizing longitudinal clinical context and structured medical knowledge that could strengthen clinical reasoning. Here, we propose a pathway to decentralized healthcare that unites multimodal wearable biosensing with multidomain AI. We review recent progress across wearable sensing modalities and summarize how multisensory fusion can improve patient profiling, enhance diagnostic discrimination, and enable earlier risk prediction. We then describe AI pipelines that integrate biosensor measurements with electronic health records and curated medical literature and knowledge graphs to support evidence‐grounded decision support. Finally, we discuss remaining challenges, including data quality and cross‐modality alignment, privacy and governance for cross‐domain data sharing, and robust generalization under real‐world heterogeneity. We highlight emerging opportunities in continual learning, retrieval‐augmented reasoning, and closed‐loop therapeutics. This “from biosignals to decisions” framework advances AI‐enabled decentralized healthcare by shifting actionable insights from the clinic into everyday environments.

## Graphical abstract

Multimodal biosensing meets multidomain AI. Wearable biosensors capture complementary biochemical and physiological signals, while cross‐device, population‐aware learning aligns noisy, heterogeneous streams. This Review distills key sensing modalities, fusion and calibration strategies, and privacy‐preserving deployment pathways that transform continuous monitoring into actionable decision support, enabling scalable decentralized healthcare beyond episodic clinic visits.

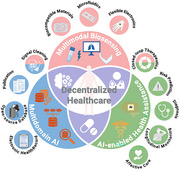

## 1 Introduction

**FIGURE 1:**
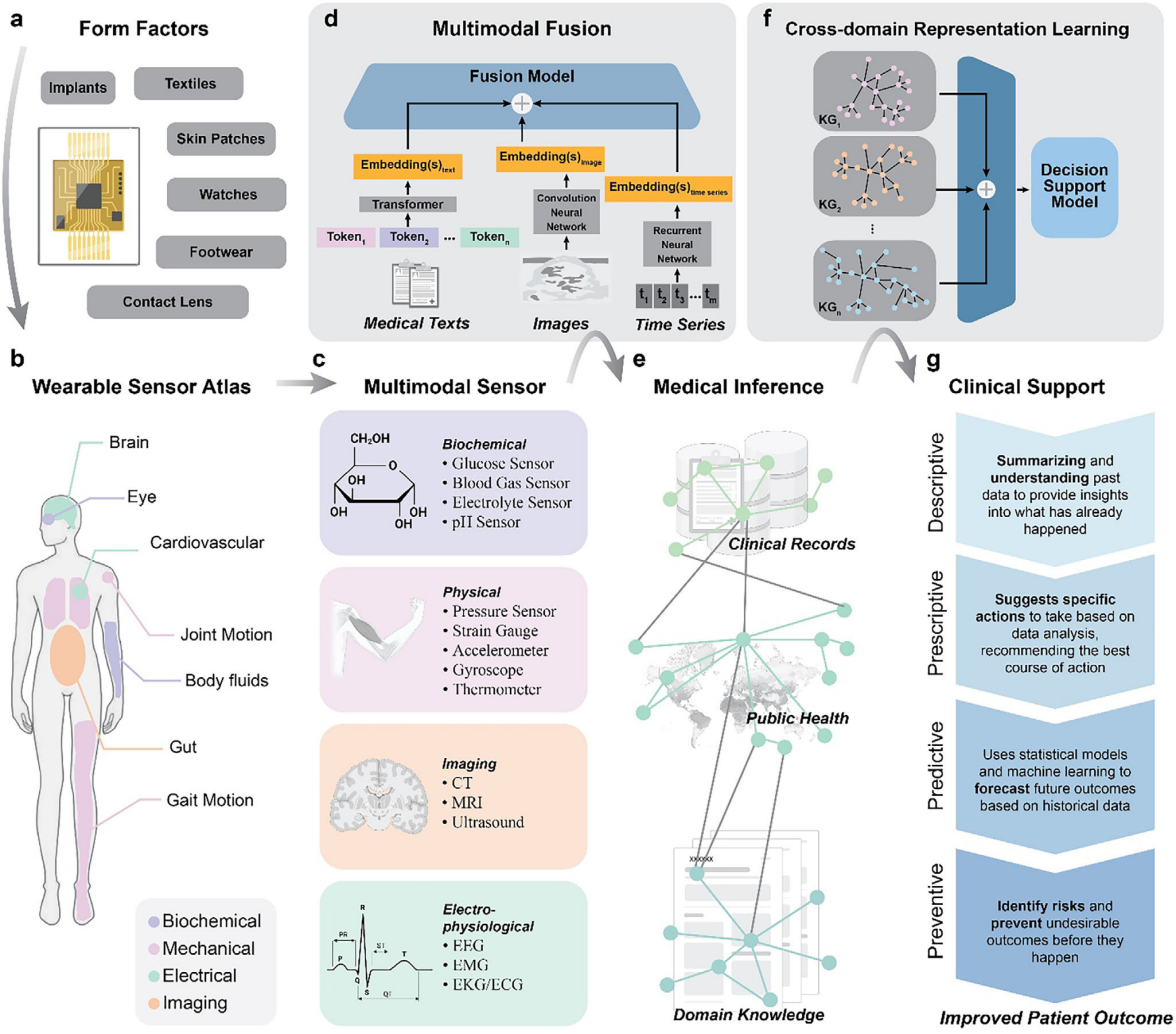
Synergy of wearable biosensors and multidomain AI. (a) Representative wearable form factors (e.g., implants, textiles, patches, watches, footwear, contact lenses) with skin‐/organ‐conformal designs. (b) Body‐site “sensor atlas” indicating anatomical targets for biosignals capture. (c) Sensing modalities accessible to wearables: biochemical, physical/mechanical, electrophysiological, and imaging. (d) Multimodal fusion pipelines aligning medical text, images, and time‐series signals into a shared representation. (e) Knowledge‐graph–based medical inference linking clinical records, public health, and domain knowledge. (f) Cross‐domain representation learning that transfers information across tasks and modalities to improve robustness in data‐sparse settings. (g) Wearables and multidomain AI enable tiered clinical decision support, from descriptive to preventive, advancing decentralized, personalized care and better outcomes.

Wearable biosensors have emerged as a platform for continuous, real‐time health monitoring, enabling non‐ or minimally invasive acquisition of physiological, biochemical, and environmental signals from diverse body sites. With miniaturized designs, tissue‐conformal form factors, and good comfort (Figure 1a), these devices embed into daily life to capture multimodal biosignals with performance approaching that of conventional clinical equipment (Figure 1b) [1, 2, 3]. Beyond point‐of‐care diagnostics, they are shifting care toward unobtrusive, decentralized, at‐home monitoring (Figure 1c), supporting continuous surveillance, earlier detection, and precision interventions while reducing clinic burden [4, 5, 6].

Despite the strong sensing performance, the bottleneck is interpretation: high‐volume, heterogeneous streams are hard to parse manually and do not scale. Multidomain AI addresses this by learning jointly from multiple modalities and injecting structured domain knowledge. Multisensory fusion (Figure 1d) aligns complementary signals into a shared representation; knowledge graphs (Figure 1e) encode curated clinical relations to strengthen inference and reduce brittleness. Cross‐domain transfer (Figure 1f) then reuses abstractions learned in data‐rich settings to inform under‐explored conditions, improving robustness when data is sparse, advancing systems from sensors to decisions. Large language models (LLMs) add a conversational layer to this stack, translating complex outputs into clear, patient‐ and clinician‐facing guidance and enabling interactive exploration of uncertainty and preferences (Figure 1g) [7, 8]. Coupled to continuous sensing, these agents move systems from raw signals to decisions, triggering, profiling risk and suggesting next steps, without requiring in‐person consultations, and thereby supporting patient self‐management while easing provider workload.

In this Review, we outline the state of the art in multimodal biosensing and multidomain AI and examine how their convergence enables decentralized, patient‐centered care. We first survey advances in multimodal wearable biosensing to illustrate complementary coverage of physiological processes and to highlight representative diagnostic and screening studies. We then focus on data fusion, showing how first‐hand sensor streams combined with electronic health records and medical literature support more accurate profiling, differential diagnosis and decision support. Finally, we discuss the remaining hurdles, standards for interoperable data integration, privacy and governance, generalizability and trust, and map opportunities for closed‐loop systems that deliver comprehensive assessment at the point of living.

## 2 Multi‐Modal Wearable Biosensors

### 2.1 From Physical Integration to Intelligent Interpretation

**FIGURE 2:**
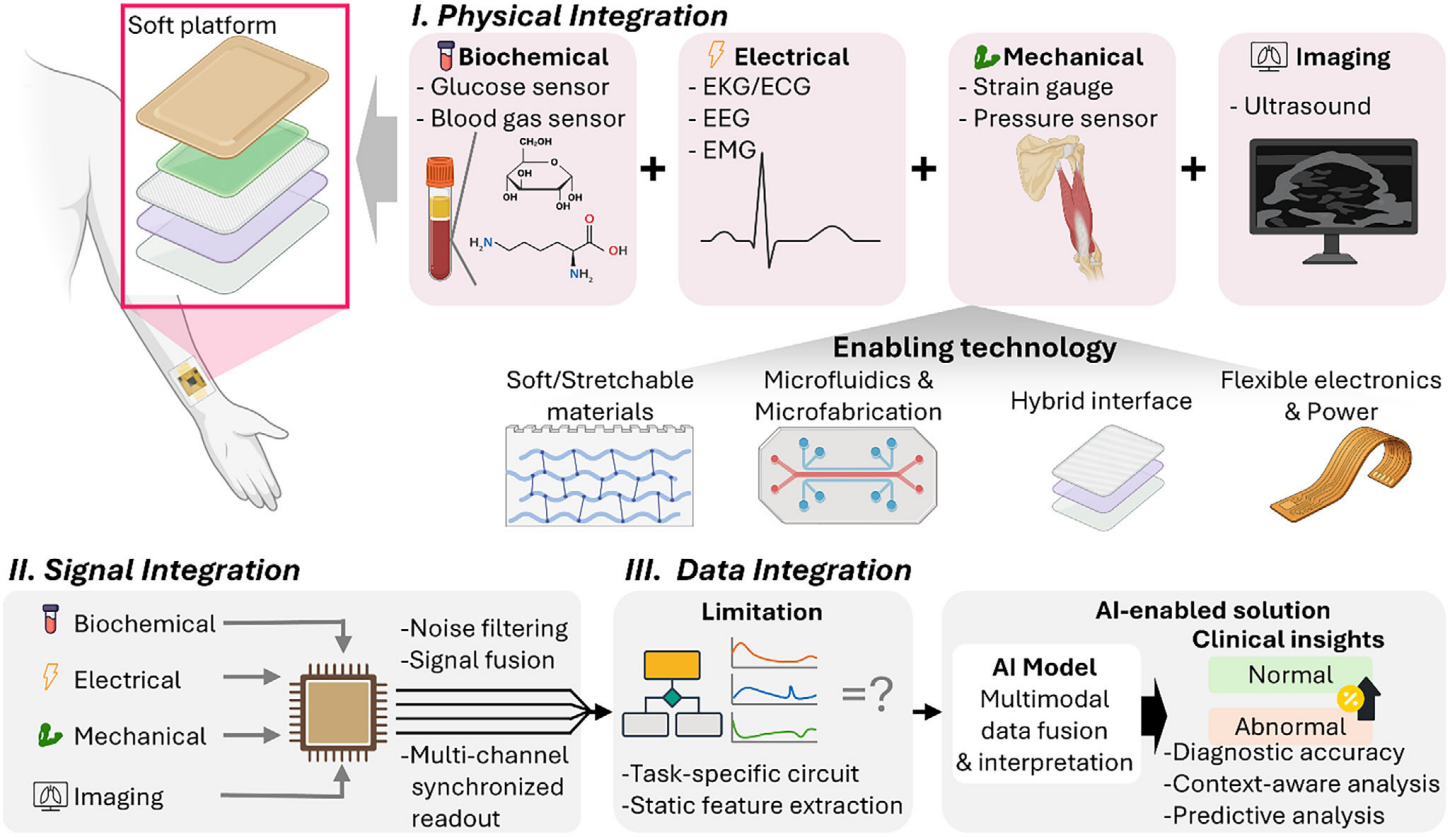
Hierarchical integration from devices to intelligence. Multimodal wearables are integrated across three tiers: *(i)* physical, co‐locating biochemical, electrical (ECG/EEG/EMG), mechanical, and imaging sensors on a soft platform enabled by stretchable materials, microfluidics, hybrid interfaces, and flexible power; *(ii)* signal, providing synchronized multi‐channel readout with noise filtering and signal fusion; and *(iii)* data, where AI‐driven fusion and interpretation deliver context‐aware, adaptive diagnostics and clinical insights.

Multimodal wearable platforms mature along three interdependent levels (Figure 2): *(* *i* *)* physical co‐location of heterogeneous sensors; *(* *ii* *)* signal‐level synchronization and shared readout; and *(* *iii* *)* data‐level fusion and interpretation. To distinguish conceptual multimodal integration from practical deployment, this hierarchy separates spatial co‐location from signal‐level synchronization and higher‐level data fusion. Accordingly, Table 1 provides objective criteria that differentiate physical sensor co‐location from true signal‐level synchronization and downstream fusion. Early multimodal wearables largely demonstrated physical co‐location of heterogeneous sensors, whereas more recent platforms increasingly enable synchronized acquisition and coordinated operation across biochemical, electrophysiological, mechanical, and imaging modalities. At the physical layer, advances in stretchable substrates, heterogeneous sensor arrays and hybrid packaging now support biochemical, electrical and mechanical sensing on a single epidermal interface. An example is the soft epidermal system by Xu et al. [9], which combines microfluidic sweat channels with iontophoretic stimulation to enable stable electrochemical, pressure, and temperature measurements during prolonged wear. The architecture carves out modality‐specific micro‐environments, using fluidic isolation for biochemical readouts and mechanical decoupling for pressure, highlighting that effective co‐location depends on controlling the interfaces each modality requires. Beyond integration complexity, physical co‐location is also motivated by the need to manage real world variability. Motion induced deformation, fluctuations in sweat rate or composition, and gradual electrode drift can substantially perturb individual sensing channels. Accordingly, many multimodal platforms deliberately incorporate mechanical, thermal, or impedance‐based reference signals at the device level to contextualize biochemical and electrophysiological readouts under dynamic, ambulatory conditions.

**TABLE 1:** Objective criteria distinguishing sensor co‐location from true synchronization in multimodal biosensing systems.

Criteria	Co‐location	True synchronization
Temporal alignment	Sensors operate with independent clocks or sampling rates; temporal proximity is assumed rather than enforced	Sensors share a common clock, time‐stamping framework, or synchronized acquisition window
Measurement context equivalence	Sensors are physically integrated but may operate under different physiological or environmental states	All modalities acquire data under equivalent physiological states, operational phases, and environmental conditions
Transport and diffusion latency compensation	Modality‐specific transport, diffusion, or tissue‐response delays are not considered	Latency arising from fluid transport, diffusion, or tissue response is explicitly modeled or compensated prior to fusion
Cross‐modal causal consistency	Apparent correlations may violate known physiological response hierarchies	Relative response timing across modalities follows physiologically plausible causal sequences
Implications for AI‐based fusion	Fusion relies primarily on feature‐level concatenation	Fusion can incorporate physiology‐aware, event‐aligned, and causality‐constrained models

*Note*: True synchronization extends beyond spatial integration and nominal temporal proximity, requiring verified alignment to shared physiological events, latency‐aware correction, and causally consistent cross‐modal relationships to ensure that multimodal signals reflect the same underlying biological process.

At the device level, maintaining meaningful synchronization over time imposes additional consistency constraints under dynamic, ambulatory conditions. Some wearable platforms address this requirement by suppressing drift through material selection, interface engineering, and controlled microenvironments [10, 11], rather than by implementing explicit periodic recalibration during wear. For example, recent physicochemical electronic‐skin systems achieve extended continuous operation (more than 100 h) with minimal signal drift by stabilizing sensor interfaces using composite material designs [9]. Similarly, bioresorbable, wireless wound‐site platforms sustain suitable electrode–tissue interfaces over weeks through conformal, biocompatible electrode architectures, enabling prolonged monitoring without disruptive device retrieval [12]. Because incorporating on‐body reference standards for continuous recalibration, such as impedance‐based self‐checks, remains challenging in many wearable form factors, synchronization is often maintained within a defined operational window in which relative drift remains acceptable by design. These examples highlight that device‐level stability is a prerequisite for reliable signal‐level synchronization in current multimodal wearable systems. As wearable form factors continue to advance, the integration of internal reference signals may offer a pathway toward periodic recalibration or self‐assessment during wear, further relaxing current constraints on long‐term synchronization.

In practice, the tiers are not cleanly separable. At the signal layer, Choi et al. [13] report an all‐printed, chipless neuromorphic patch that couples biochemical, mechanical and thermal sensing with on‐board analogue computation, directly interpreting multimodal inputs to achieve end‐to‐end transduction, from molecular recognition to feature classification. The elegance is offset by trade‐offs: task‐specific analogue readouts and fixed feature maps constrain portability to new analytes or physiological contexts.

These constraints motivate AI‐level integration, in which multimodal streams are fused and interpreted by machine‐ or deep‐learning models rather than hard‐wired signal paths. Wang et al. [14]. exemplify this transition with a chronic‐wound microfluidic platform that measures pH, uric acid, temperature and impedance, then classifies wound status and tracks healing via a trained model. Embedding inference within the sensing workflow shifts devices from passive monitoring to adaptive, clinically actionable insight. Such developments establish the AI tier as the apex of multimodal biosensing, transforming patches into context‐aware, self‐improving diagnostic systems. Priorities now include compact edge‐AI processors, energy‐efficient neuromorphic hardware and interoperable data standards to orchestrate heterogeneous sensors, ultimately enabling intelligent, closed‐loop multimodal monitoring from sensors to decisions for decentralized healthcare.

### 2.2 Cross‐Modality Synergies and Applications

The emphasis is shifting from structural co‐location to functional interdependence, where heterogeneous sensors actively condition one another to reveal coupled physiology. Instead of operating as isolated modules, biochemical, electrical, mechanical and imaging modalities exchange contextual cues in real time, yielding cross‐modal correlations that capture feedback loops, neuromuscular coupling, electro‐metabolic regulation and mechano‐chemical transduction. Here, multimodal breadth is defined by coverage across distinct sensing domains rather than by the number of parameters measured within any single modality. To operationalize this definition, Table 2 summarizes representative wearable platforms spanning orthogonal sensing domains, illustrating how breadth arises from domain‐level coverage rather than parameter multiplicity. This coordination enables the detection of composite, multi‐energy phenomena that no single sensor can resolve, advancing systems from sensors to decisions. We highlight four representative axes of synergy below. Importantly, these synergies arise not simply from sensors coexisting on the same platform, but from deliberate synchronization strategies, such as shared timing architectures, unified readout electronics, and coordinated sensing cycles. These designs promote temporal alignment across modalities, enabling reliable real time extraction of cross domain relationships rather than post hoc inference.

**TABLE 2:** Domain‐based taxonomy of multimodal wearable sensing systems and representative sensing targets.

Multimodal domain	Sensing targets	Representative platform/device type	Ref.
Biochemical‐Electrical	Glucose, ECG	Microfluidic epidermal sweat patch integrating ECG electrodes with wireless readout	[15]
	Glucose, heartrate, pH, temperature	Stretchable skin‐mounted patch combining electrochemical sensors, biopotential, and thermal sensing	[16]
	Glucose, lactate, uric acid, temperature, pH, ammonium	Microfluidic sweat‐analysis patch with an integrated electrochemical sensor array	[18]
	Biofluid analytes	Microfluidic electrode‐integrated platform emphasizing multimodal electrode design	[257]
Mechanical‐Imaging	Tissue elasticity (ultrasound shear‐wave dynamics)	Wearable ultrasound elastography patch with active mechanical excitation and imaging transducers	[19]
	Tissue stiffness (mechano‐acoustic elastography)	Wireless wearable elastography system based on mechano‐acoustic wave sensing	[20]
Mechanical‐Optical	Wrist pulse waveform, radial artery diameter change, skin vibration	Optoelectronic hybrid microfiber with LPG	[258]
Mechanical‐Thermal	Strain, pressure, temperature	Twisted multifunctional fiber enabling simultaneous mechanical and thermal sensing	[259]
Mechanical‐Electrical	Muscle structure (ultrasound imaging) and activation (EMG)	Wearable ultrasound–EMG integrated patch for dynamic muscle assessment	[260]
	ECG, EMG, joint/motion signals, tactile pressure	Liquid‐metal–based skin‐mountable multimodal sensor system	[261]
Tri‐modal (Optical‐Mechanical‐Physiological)	Swelling, temperature, humidity, SpO_2_	Epidermal patch integrating optical oximetry, strain sensing, and environmental sensors	[28]
Tri‐modal (Electrochemical‐Electrical‐Thermal)	Interstitial glucose, lactate, ECG, temperature	Fully printed hybrid system: implantable electrochemical biosensors with wearable ECG and temperature sensing	[262]
	Core body temperature, ECG/HR, sweat rate	Probe‐style wearable system integrating core temperature, ECG, and sweat sensing	[263]
	Sweat biomarkers, biomechanical stress, skin temperature	Multimodal wearable platform for biostress monitoring and sweat analysis	[264]
	Sweat glucose, lactate, uric acid, pH, skin impedance, temperature	Skin‐integrated bionic sensor patch for biochemical and biophysical monitoring	[265]
Tri‐modal (Electrochemical‐Electrical‐Mechanical)	Glucose, alcohol, pressure/touch, activity context	Self‐powered wearable sensor combining biofuel‐based electrochemical sensing with motion‐coupled mechanical inputs	[266]
Tri‐model (Electrochemical‐Mechanical‐Thermal)	Chemical species, temperature, flow/strain	Multimodal sensor system for coupled chemical and physical monitoring	[267]
Tri‐model (Electrical‐Mechanical‐Thermal)	Bioelectrical signals, strain, temperature	Soft multifunctional electronic material platform	[268]
Tri‐modal (Electrical‐Thermal‐Optical)	Arterial pulse waveform, skin temperature, UV/NO_2_	Wrist‐worn multimodal band using heterojunction sensors with ML‐based signal decoupling	[269]
Tri‐Modal (Electrical‐Optical‐Mechanical)	ECG, body movement, PPG, hemodynamic, temperature	Vialess heterogeneous skin patch combining bioelectrodes, optical sensors, and inertial and thermal modules	[29]

Abbreviations: SpO_2_, oxygen saturation; ECG, electrocardiogram; PPG, photoplethysmography; LPG, long‐period grating; ML, machine learning; UV, ultraviolet.

Beyond enhancing physiological insight, coordinated acquisition can also improve robustness under real world conditions. By correlating biochemical, electrical, mechanical, and imaging signals collected concurrently, multimodal systems can better distinguish true physiological changes from artefacts arising from motion, variable sweat composition, or sensor drift, which often confound single modality measurements. Although presented in specific clinical contexts, these case studies should be viewed as system level templates rather than disease specific solutions. The core sensing, fusion, and decision loop can be transferred to other applications by substituting domain relevant biomarkers, models, and clinical objectives while preserving the underlying multimodal architecture.

#### 2.2.1 Biochemical and Electrical Synergy

**FIGURE 3:**
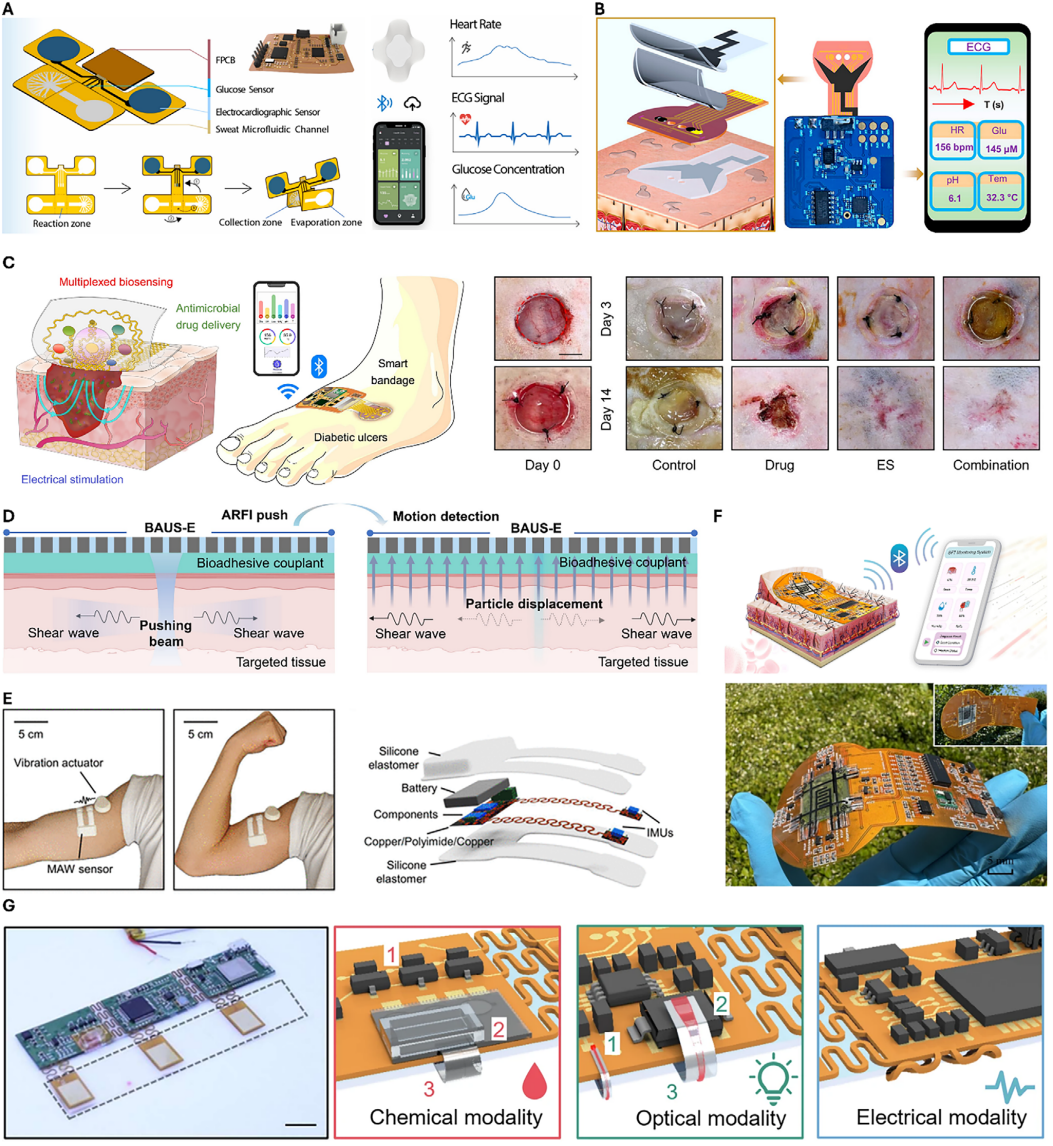
Cross‐modality synergies in multimodal wearable biosensing. (A) Biochemical–electrical hybrid patch that co‐integrates a glucose sensor and electrophysiology on a flexible microfluidic platform for simultaneous metabolic and cardiac monitoring. Reproduced with permission [15]. Copyright 2022, Elsevier. (B) Microfluidic wearable combining sweat metabolite analysis, ECG, and temperature sensing in a single soft architecture to reveal electro‐metabolic correlations. Reproduced with permission [16]. Copyright 2023, American Chemical Society. (C) Stretchable, wireless bioelectronic dressing for infected‐wound care coupling multiplexed biochemical sensing (pH, glucose, uric acid), thermal monitoring, and electrical stimulation for closed‐loop therapy. Reproduced under the terms of the CC BY 4.0 license [18]. Copyright 2023, AAAS. (D) Bioadhesive ultrasound elastography patch uniting active mechanical excitation with imaging for continuous, wearable mapping of tissue elasticity. Reproduced under the terms of the CC BY 4.0 license [19]. Copyright 2024, AAAS. (E) Wireless mechano‐acoustic elastography platform synchronizing strain sensing with shear‐wave imaging to track tissue mechanics during movement. Reproduced under the terms of the CC BY 4.0 license [20]. Copyright 2025, AAAS. (F) Multimodal skin‐flap monitor integrating mechanical (strain, temperature), optical (SpO_2_), and humidity sensing for quantitative assessment of graft perfusion and viability. Reproduced with permission [28]. Copyright 2025, American Chemical Society. (G) Tri‐modal soft patch that merges chemical, optical, and mechanical modalities within one platform to enable self‐regulation, context‐aware epidermal electronics Reproduced under the terms of the CC BY 4.0 license [29]. Copyright 2025, Springer Nature.

The joint acquisition of biochemical and electrical signals enables concurrent tracking of metabolic dynamics and electrophysiological state within a single platform, bridging molecular and ionic domains. This coupling offers a direct view of electro‐metabolic feedback, in which biochemical shifts modulate electrical excitability and vice versa. Li et al. [15] report a conductive hydrogel–paper hybrid patch that records ionic and electrophysiological signals through one soft interface (Figure 3a). The hydrogel serves both as an ion‐transport matrix for Na^+^, K^+^, lactate and pH sensing and as a biocompatible electrode for surface biopotentials (ECG/EMG‐type). Whereas stand‐alone electrochemical or electrophysiological sensors cannot readily disentangle metabolic drift from changes in electrode–skin impedance, this hybrid design correlates ionic imbalance with tissue excitability in real time. The result is a self‐calibrating bioelectric readout and direct observation of electrolyte‐driven neuromuscular fatigue, illustrating how cross‐modal coupling elevates routine signal tracking to integrated physiological interpretation.

Extending this concept, Zahed et al. [16] integrate microfluidic sweat analysis, ECG and temperature sensing into a single flexible patch (Figure 3b). The microfluidic network stabilizes sweat collection and mitigates evaporation artifacts, enabling continuous Na^+^, K^+^ and lactate measurements, while co‐located ECG electrodes capture autonomic responses to metabolic and thermal stress. Correlating these heterogeneous streams reveals electro‐metabolic coupling, for example, heart‐rate acceleration accompanied by ionic imbalance, thereby improving signal fidelity and enabling context‐aware assessment of systemic homeostasis [17].

Moving from monitoring to intervention, Sani et al. [18] present a stretchable, wireless bioelectronic system for infected‐wound care that unites multiplexed biochemical sensing (pH, glucose, uric acid), temperature monitoring and closed‐loop electrical stimulation (Figure 3c). Temperature functions as both an inflammatory marker and a control parameter for electro‐therapeutic output, allowing the device to differentiate infection stages and autonomously adjust stimulation to accelerate tissue repair while suppressing bacterial growth. This architecture fuses diagnosis and therapy within a single conformable form factor.

Despite these advances, most biochemical‐electrical platforms remain surface‐confined, capturing epidermal or superficial activity without resolving subsurface mechanics or perfusion that often govern electro‐metabolic shifts. Progress will require integration with mechanical and imaging modalities to situate biochemical‐electrical signals within a spatial and structural context.

#### 2.2.2 Mechanical and Imaging Synergy

Mechanical sensors (*e.g*., strain gauges and pressure arrays) capture the temporal dynamics of deformation but offer little anatomical depth. Imaging modalities, such as ultrasound elastography and tomographic technique, reveal internal structure and stiffness, but are often limited by rigid form factors, operator dependence, and low frame rates. Fusing these streams allows wearable systems to quantify how deformation and stiffness co‐evolve, yielding richer insight into musculoskeletal, vascular, and broader mechano‐physiology. Liu et al. [19] demonstrate mechanobiological synergy by integrating active mechanical excitation with ultrasound visualization in a single adhesive patch (Figure 3d). Acoustic radiation force generates controlled shear waves, and co‐registered ultrasound tracks their spatiotemporal propagation to compute elasticity maps. By embedding both actuation and imaging in a conformal device, the system enables automated, long‐duration elastography of in vivo organs, such as 48‐h liver monitoring, converting ultrasound from a predominantly structural tool into a functional, mechanobiological modality.

Extending this approach, Li et al. [20] report a wireless mechano‐acoustic elastography patch that synchronizes high‐bandwidth surface‐strain sensing with depth‐resolved shear‐wave imaging (Figure 3e). The system synchronized high‐bandwidth mechanical deformation data with depth‐resolved stiffness maps, enabling continuous tracking of joints and soft tissues under active motion. By capturing both surface kinematics and subsurface mechanical responses within a unified framework, the platform revealed how externally measured strain relates to internal stress propagation. This synergy enables a single patch to co‐measure surface deformation and depth‐resolved shear modulus, mapping observable motion onto internal stress, an insight unattainable by either modality alone, with applications in rehabilitation (tracking joint kinetics and muscle stiffness) [21, 22, 23], cardiovascular monitoring (arterial deformation and load‐dependent stiffness) [24, 25], and organ health (progressive hepatic or renal stiffening) [19, 26, 27]. Looking ahead, real‐world use cases will benefit from tri‐ or multimodal platforms that integrate mechanics and imaging with additional sensing dimensions to achieve adaptive, context‐aware physiological mapping.

#### 2.2.3 Other Multi‐Modal Synergy

Standard postoperative surveillance of skin flaps often depends on a single indicator, temperature, color or perfusion, each offering a partial view of viability. Temperature reflects superficial flow but not oxygenation; strain gauges report suture tension yet miss perfusion deficits; optical photoplethysmography (SpO_2_) quantifies oxygen saturation but degrades with swelling or motion.

To address these gaps, Luo et al. [28] introduce a conformal thin‐film patch that integrates mechanical (strain, temperature), SpO_2_ and humidity sensing (Figure 3f). The device simultaneously tracks perfusion, oxygenation and graft tension in real time; humidity sensing captures vapor and exudate dynamics, while temperature and strain channels quantify thermomechanical stability. By correlating these heterogeneous inputs, the system detects early vascular compromise that would evade any single modality, shifting flap monitoring from qualitative inspection to quantitative, multimodal mapping. In a related advance, Lee et al. [29] present a wireless heterogeneous skin patch unifying chemical, optical and mechanical modalities on a single soft platform (Figure 3g). Micro‐reservoir‐free chemical sensors measure ionic and metabolic analytes; optical elements enable photoplethysmography and fluorescence readout; stretchable strain sensors provide motion context. This tri‐modal architecture enables simultaneous analysis of biochemical composition, optical perfusion and mechanical deformation, revealing cross‐domain interactions such as strain‐induced perfusion shifts or metabolically driven optical changes. Beyond passive sensing, integrated local stimulation supports closed‐loop chemical–optical–mechanical feedback, pointing to self‐regulating, context‐aware epidermal electronics that emulate the integrative responsiveness of skin.

### 2.3 From Multimodal Sensing to Integrated Interpretation

Despite rapid progress, seamless integration across heterogeneous sensing modalities remains difficult, with obstacles spanning materials, electronics and, increasingly, information science. These challenges arise not only from mechanical mismatch, power management and long‐term stability, but also from the complexity and heterogeneity of multimodal data, which impose new constraints on interpretation, reliability and scalability [25, 30, 31, 32].

At the device level, multimodal platforms must balance biocompatibility, stability and signal isolation. Each modality operates under distinct physicochemical and temporal regimes [33, 34]. In real world use, wearable systems are further challenged by motion artefacts, inter and intra subject variability in sweat composition, and gradual electrode drift caused by fouling or surface reconstruction. Multimodal designs can partially mitigate these effects by providing contextual reference channels, such as motion, strain, or temperature, and by enabling cross modal consistency checks. In parallel, AI based models can learn subject specific baselines and distinguish physiological trends from measurement instability over time. Architectures must prevent electrochemical interference, mechanical cross‐talk and hydration loss while preserving skin conformity, constraints that enforce persistent engineering trade‐offs [35, 36]. Continued advances in materials, microfabrication and packaging are mitigating many of these physical limitations, but they do not remove the core integration problem [37, 38].

The more fundamental bottleneck now lies in data interpretation. Wearables generate unprecedented volumes of physiological signals, from heart rate, sleep and ECG to oxygen saturation and glucose, yet most systems still analyze each stream in isolation [32, 39]. This fragmentation reflects a broader limitation of medical AI, which often tackles narrow, single‐task objectives rather than modelling health as a continuous, interconnected state.

Realizing the full potential of multimodal biosensing will require intelligent interpretive frameworks. AI offers a means to learn latent correlations across heterogeneous streams and to dynamically link molecular, electrical, mechanical and behavioral signals to physiological meaning. Recent multimodal and attention‐based architectures illustrate how static and time‐dependent inputs can be fused to yield interpretable, patient‐specific forecasts [40, 41]. In this view, the future of multimodal biosensing will be defined less by adding sensors and more by making sense of them, transforming raw, fragmented biosignals into coherent, context‐aware representations of human health.

## 3 Multidomain AI

While wearable biosensors excel at high‐fidelity, real‐time sensing, their utility is constrained without advanced interpretive intelligence. In the absence of a framework that synthesizes heterogeneous inputs, raw biosignals remain underexploited for decentralized healthcare. Multidomain AI addresses this gap by learning across modalities and knowledge sources, transforming isolated sensor streams into actionable medical intelligence [42, 43]. In addition to aggregating information from diverse data types and settings, it transfers knowledge between domains, improving generalization and robustness [44, 45]. Integrated with first‐hand indicators from wearable biosensors, multidomain AI can form a more accurate picture of an individual's health state; paired with Electronic health records (EHRs), it supports downstream diagnosis and risk prediction grounded in contemporaneous physiology. Equipped with cross‐domain priors, modern AI agents can generate professional, verifiable textual and visual outputs, and, via conversational interfaces, engage in iterative dialogues that elicit preferences and constraints, providing clearer, more personalized guidance [46, 47]. Real‐time fusion further enables immediate feedback, supporting timely treatment planning and healthier behavioral choices. Ultimately, decentralized healthcare will hinge less on adding sensors than on unifying their signals into coherent, user‐centric decisions.

Although AI is reshaping diagnostics [48, 49, 50], treatment planning [51, 52], precision medicine [53, 54], surgery assistance [55, 56], and disease prediction [57, 58], most deployed systems remain narrow, excelling at single tasks without transferable reasoning. The next stage centers on multidomain AI capable of applying knowledge and logic across disciplines to solve complex, clinically grounded problems. A core enabler is multimodal fusion: the joint representation of text, images, video and time‐series biosignals to construct comprehensive patient embeddings [59, 60]. Analogous to human perception, fusion captures nuances that single modalities miss, yielding a more holistic clinical profile. In healthcare, such models can improve understanding of health trajectories [32, 61], personalize treatments [62, 63], and unlock more sophisticated applications [64, 65].

Complementing fusion is cross‐domain representation learning (CDRL), which aligns concepts across domains so that insights transfer from one context to another [66, 67]. In medicine, where decisions depend on structured knowledge and causal reasoning, CDRL helps models recognize abstract patterns, tolerate missing or ambiguous data, and reuse strategies learned from related diseases or subtypes [68]. Working in tandem, multimodal fusion and CDRL support the inference of appropriate interventions for novel cases from accurate, multimodal descriptions of physiological state, much as clinicians draw on prior cases and the literature [69, 70].

**FIGURE 4:**
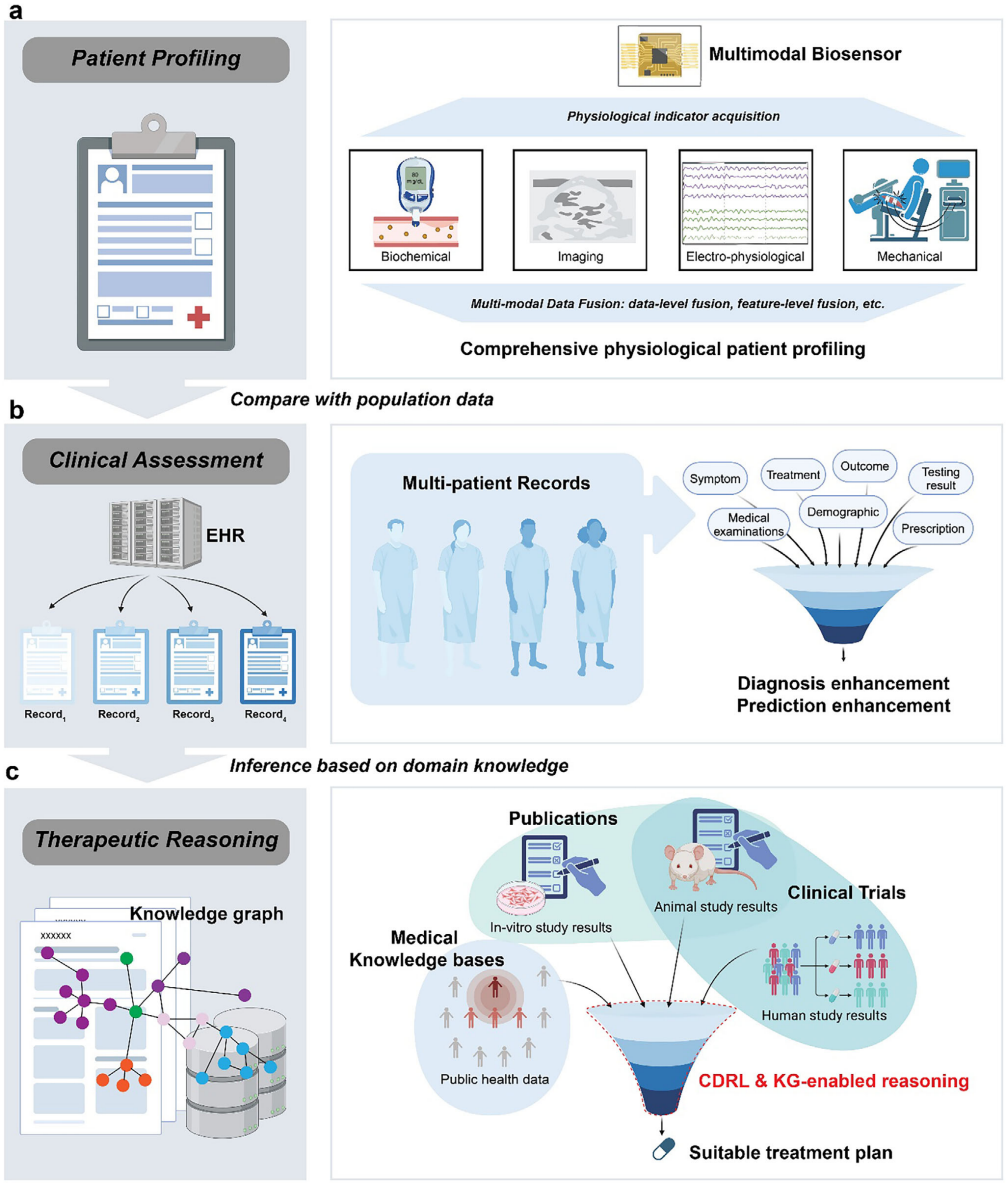
Multidomain AI workflow in healthcare. (a) Patient profiling: Multimodal physiological data (biochemical, imaging, electrophysiological, mechanical) are fused at the data/feature level to build a comprehensive profile. (b) Clinical assessment: The profile is benchmarked against population‐scale EHRs, spanning symptoms, exams, demographics, treatments, outcomes, and prescriptions, to enhance diagnosis and prediction. (c) Therapeutic reasoning: Domain knowledge graphs assembled from publications, public‐health databases, and clinical‐trial results support cross‐domain representation learning and reasoning to recommend suitable treatment plans.

A fully integrated, multidomain‐AI healthcare stack comprises three interconnected layers: *(* *i* *)* Patient profiling: constructing dynamic physiological representations from the sensing technologies (Figure 4a); *(* *ii* *)* Clinical assessment: diagnostic and prognostic models that operate on first‐hand biomarkers to stratify risk and forecast outcomes (Figure 4b); *(* *iii* *)* Therapeutic reasoning (Figure 4c): decision models that propose, sequence and adapt treatments under domain knowledge and patient‐specific constraints.

### 3.1 Patient Profiling

Comprehensive patient profiling is central to personalized care. Biosensing modalities yield complementary views of physiology but considered in isolation they produce incomplete or noisy portraits of health. Multimodal fusion addresses this limitation: by encoding heterogeneous signals into a shared latent space, models consistently outperform unimodal baselines across diverse tasks and reduce population risk through complementary information capture [71, 72].

**FIGURE 5:**
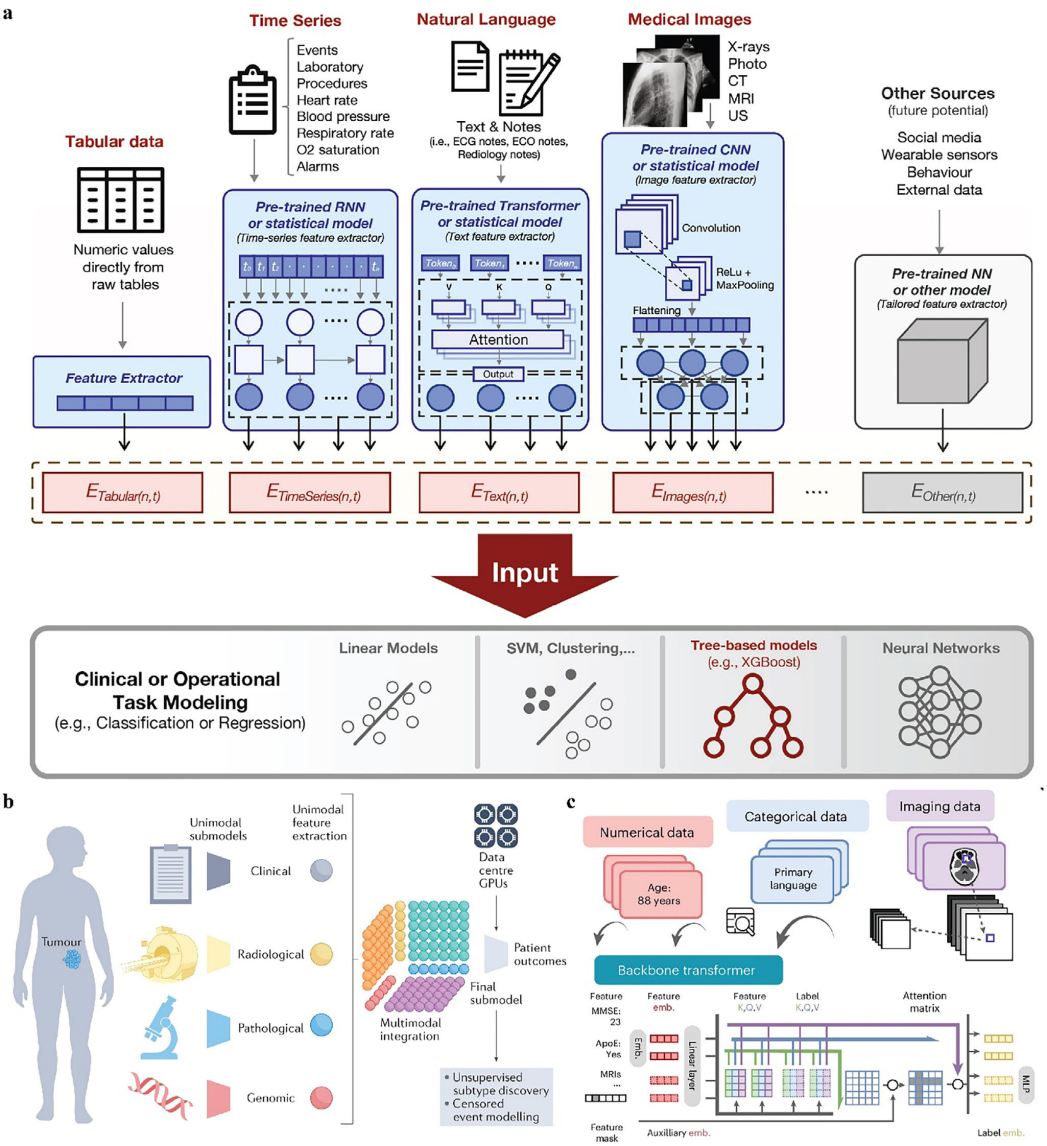
Multi‐inter‐modal medical fusion models. (a) The holistic AI in medicine (HAIM) framework unifies tabular, time‐series, text, and image streams via modality‐specific feature extractors (e.g., Recurrent neural network (RNN)/Transformer/ Convolutional neural network (CNN) or statistical models) to produce embeddings that feed downstream classifiers/regressors (linear, SVM/cluster, tree‐based, neural nets). Reproduced with permission [94]. Copyright 2022, Springer Nature. (b) A multimodal prognosis pipeline mitigates data sparsity by coupling unimodal submodules (clinical, radiological, pathological, genomic) with late‐stage integration to yield intermodal features for outcome prediction and subtype discovery. Reproduced with permission [254]. Copyright 2022, Springer Nature, permission pending. Sub‐models extract unimodal features from different data modalities, followed by multimodal integration to produce intermodal features. (c) Differential dementia diagnosis using a transformer backbone that ingests numerical, categorical, and imaging inputs; each feature is embedded with a modality‐aware scheme before cross‐feature attention and prediction. Reproduced with permission [105]. Copyright 2024, Springer Nature.

Clinicians already synthesize multiple indicators in practice, yet even expert judgment can miss subtle, high‐dimensional patterns. Multimodal AI extends this reasoning by jointly analyzing disparate streams and uncovering interactions that may elude human assessment (Figure 5a), improving diagnostic accuracy and efficiency for truly personalized care [73, 74, 75]. In time‐critical settings, such integration enables rapid, expert‐level support, for example, fusing abdominal radiographs with laboratory data for surgical decision‐making in necrotizing enterocolitis [76, 77], or combining white‐light and weak‐magnification endoscopy for real‐time gastric neoplasm diagnosis [78, 79].

Rich profiles also require context beyond first‐hand sensor data. Demographics, environment and behavior shape physiological readouts; integrating these second‐hand factors with biosignals yields holistic representations that link structure, function and context, enabling more precise diagnoses and tailored interventions. Multimodal fusion typically increases diagnostic confidence and robustness over unimodal approaches [61, 80], and, when paired with attention or concept‐bottleneck designs, can improve interpretability for clinicians [81, 82] (Figure 5b). As sensing technology advances, these integrated profiles will further improve personalized care.

Two complementary fusion strategies are used: *(* *i* *)* Intra‐modal fusion enhances information within a single modality. At the data level, techniques such as multi‐image source fusion merge pixels from multiple views or focal planes to improve clarity [83, 84]. Multi‐focus image fusion (MFIF) combines images captured at different depths into a sharper composite; applied to pathological sections across 30 cancer types, MFIF improved downstream segmentation, classification and pre‐diagnosis tasks [85, 86]. Intra‐modal fusion can also combine related imaging types (*e.g*., MRI with PET) to boost disease classification accuracy, as shown for Parkinson's disease [87, 88]; *(* *ii* *)* Inter‐modal fusion integrates heterogeneous sources (*e.g*., signals, images, text) to construct more complete and actionable patient representations [89]. Feature‐level fusion is particularly effective when data are sparse or heterogeneous, as in precision oncology [84]. In practice, frameworks such as MedFuse combine clinical time‐series data with chest radiographs to improve in‐hospital mortality prediction and phenotype classification, outperforming sequence‐only baselines that miss visual cues [90]. In neurodegeneration, fusing MRI with EEG enhances Alzheimer's diagnosis relative to either modality alone [91], while combining laboratory tests with MRI segmentation further improves predictive performance [92]. In oncology, joint analysis of dynamic contrast‐enhanced MRI and digital mammography yields superior breast‐lesion classification compared with single‐modality models [93].

As fusion methods mature, patient profiling will move from siloed measurements to unified, context‐aware embeddings that support precise, personalized decisions. Integrating unimodal and multimodal pipelines at the data and feature levels allows complementary strengths to compensate for individual weaknesses, translating complex physiological streams into actionable clinical insight.

### 3.2 Clinical Assessment

Clinical assessment aggregates individual profiles at the cohort level to uncover shared risk factors, trajectories and treatment responses. EHRs curate longitudinal, multimodal data, diagnoses, therapies, medications and observed responses, at varying granularities. Mining these histories enables discovery of symptom–treatment–outcome correlations that sharpen differential diagnosis when presentations overlap [94, 95], and supports prognostic modelling of disease progression and complications [96, 97].

However, discrepancies between real time biosensors derived patient profiles and retrospective EHR patterns are inevitable in decentralized and ambulatory settings. Such conflicts are increasingly recognized as informative rather than erroneous and are commonly addressed through uncertainty aware multimodal fusion and clinician in the loop workflows rather than automatic override [32, 94, 98, 99]. In practice, deviations between contemporaneous physiological signals and historical clinical expectations are explicitly detected, weighted by signal quality, recency, and contextual metadata, and escalated for human adjudication when clinically significant. This workflow positions multidomain AI not as a mechanism for replacing clinical records with sensor data, but as a contextual reasoning layer that aligns firsthand physiological measurements with longitudinal medical knowledge to support safe, interpretable, and accountable clinical decision making.

#### 3.2.1 Diagnostic Enhancements

When a patient's characterization aligns with disease profiles encoded in EHRs, AI can make knowledge‐enhanced, evidence‐based diagnoses [99, 100]. As longitudinal records accumulate, fusing these histories with an accurate, contemporaneous representation of physiological state from patient profiling further enhances diagnostic confidence (Figure 5c).

Imaging‐centric diagnosis primarily benefits from EHR fusion across classification, detection and causal attribution. For classification, Tang et al. [101] propose FusionM4Net, a two‐stage multimodal learner for multi‐label skin disease. Stage one integrates clinical and thermoscopic image embeddings; stage two augments the dataset with patient metadata (age, sex, history) to train a support‐vector machine, achieving 78.5% accuracy and underscoring the gains from adding structured context. In histopathology, Marini et al. [102] embed fine‐grained textual descriptors into image representations for >6,000 whole‐slide colon images, improving tissue classification over unimodal baselines and highlighting the value of tight image–text coupling.

For detection, Huang et al. [103] target pulmonary embolism with a model that fuses imaging with EHR features, reaching an area under the receiver operating characteristic curve (AUROC) of 0.947 and outperforming single‐modality approaches, an advance with clear implications for timely intervention and resource allocation. Multimodal reasoning can also aid diagnostically ambiguous presentations: Chen et al. [104] report a fused model for fever of unknown origin, illustrating how combined signals improve inference under uncertainty. Beyond recognition, multimodal integration helps uncover aetiologia. Xue et al. [105] combine demographics, histories and medication records with neuroimaging and neuropsychological tests to differentiate dementia subtypes, enabling more precise, subtype‐specific management. Similarly, Liang et al. [106] introduce LungDiag, which leverages clinical representations from EHRs to support diagnosis across diverse respiratory diseases, extracting salient features to streamline clinician decision‐making.

#### 3.2.2 Prediction Enhancements

EHRs provide longitudinal, multimodal traces of patient state that enable forward inference, identify risk factors, stratifying individuals by likelihood of deterioration and guide timely intervention [107, 108]. Predictive modelling thus informs both routine and time‐critical care. In non‐urgent care, accurate forecasts support proactive, personalized management. For chronic disease, analytics can flag high‐risk patients and anticipate complications, enabling earlier lifestyle, pharmacologic or monitoring interventions that reduce hospitalizations [109, 110]. For example, Alloghani et al. [111] analyzed multi‐hospital encounter data (demographics, diagnoses, and laboratories results) to identify readmission predictors in diabetes, while Li et al. [112] proposed a framework that fuses multimodal clinical data and demographics to learn patient‐specific representations, improving next‐diagnosis prediction.

Beyond structured fields, EHR corpora include clinician–patient dialogues that encode valuable context. Zheng et al. [113] introduced an EHR‐driven, multimodal knowledge‐graph attention model for COVID‐19 that integrates CT, radiography and ultrasound with conversational data to enhance complication‐risk identification. In emergencies, minutes matter. Real‐time decision‐support models that monitor streaming physiology and EHR context can pre‐alert clinicians to impending instability (e.g., septic shock, cardiac arrest), enabling rapid evaluation and intervention. Kim et al. [114] demonstrated a proof‐of‐concept EHR‐based model that predict severe postoperative complications up to 4 h before critical events, highlighting the value of coupling sensor data with records for bedside alerts. Likewise, Lyu et al. [115] showed that combining structured EHR fields with clinical notes significantly improves mortality prediction relative to structured‐only baselines.

EHR‐powered prediction enables early risk identification, tailored care plans and prompt intervention across acuity levels [116, 117]. By leveraging structured variables, free‐text narratives and complementary clinical modalities, these models increase accuracy and clinical utility, supporting a more anticipatory, data‐driven healthcare system.

### 3.3 Therapeutic Reasoning

Clinical reasoning is inherently integrative: clinicians synthesize symptoms, histories, examinations, laboratory findings and contextual factors (lifestyle, environment) to generate differential diagnoses, estimate outcomes and tailor therapies [118, 119]. This process blends objective evidence with experiential judgement accumulated over years of training.

As data volume and complexity grow, computational approaches can augment this reasoning [120, 121]. Knowledge graphs (KGs) encode medical entities, symptoms, diagnoses, drugs, procedures, and their relations across specialties, enabling CDRL that transfers insights (for example, from cardiology to endocrinology) and surfaces patterns not readily apparent to humans [122, 123]. Embedded within AI pipelines, KGs support diagnosis prediction, treatment recommendation and personalized care planning, improving clinical decision support by grounding in structured medical knowledge [124, 125]. This workflow follows: (*i*) curate and align multimodal patient signals with KG concepts; (*ii*) perform CDRL over the graph to infer latent, patient‐specific representations; and (*iii*) generate therapy proposals that respect evidence, comorbidities and clinician constraints.

#### 3.3.1 Knowledge Graph (KG) and Cross‐Domain Representation Learning (CDRL)

Effective reasoning depends on data coverage. Common chronic diseases enjoy abundant examples, whereas rare or emergent conditions suffer scarcity. Multimodal fusion can compensate for missing signals by aligning what is available across sources.

Beyond empirical intuition, the reliability of cross‐domain transfer learning has been systematically evaluated in time‐series settings. In a large‐scale study spanning six within‐domain and twenty‐four cross‐domain source–target pairs across seismological, speech, medical, and financial signals, pretrained models achieved statistically significant improvements over training from scratch and converged more rapidly [126]. These findings indicate that transferable representations can be learned even across seemingly unrelated signal domains, reducing data requirements and enabling effective reuse with minimal fine‐tuning. Liu et al. [98] show that combining structured biomarkers with unstructured clinical notes mitigates modality gaps, allowing knowledge learned from well‐represented cohorts to transfer to under‐represented or novel cases. Yu et al. [67] introduced a hybrid multimodal fusion framework trained on MRI and PET data from the Alzheimer's disease neuroimaging initiative (ADNI) database, a comprehensive collection of longitudinal multimodal clinical data for Alzheimer's disease, and showed that it can be transferred to distinct, smaller cognitive‐decline cohorts with minimal additional retraining to forecast progression trajectories. This result illustrates practical cross‐cohort reuse of multimodal embeddings.

KGs further narrow data and knowledge gaps by encoding clinically meaningful relations, drug–drug interactions, symptom–disease links, treatment–outcome dependencies, and supporting inference when specific details are absent [127, 128]. By capturing both explicit and implicit connections, KGs enable models to reason over incomplete records. Building on this idea, Gong et al. [129] propose a safe medicine recommendation (SMR) framework that integrates EMRs (Medical Information Mart for Intensive Care (MIMIC)‐III) with medical KGs (ICD‐9 ontology, DrugBank) to construct a heterogeneous graph of patients, diseases and medications. Learned embeddings in a shared space cast therapy selection as link prediction while accounting for diagnoses and potential adverse reactions.

CDRL leverages domain KGs to improve both performance and explainability. Bean et al. [130] predict unknown adverse drug reactions using a KG spanning drugs, targets, indications and reactions, accurately classifying known events and anticipating unseen risks. Chandak et al. [131] introduce PrimeKG, a multimodal graph linking >17,000 diseases to processes, pathways, anatomy, phenotypes and drug–symptom interactions, providing mechanistic context for decision support. With such structured priors, domain‐specialized foundation models become more capable: Tu et al. [132] present Med‐PaLM Multimodal, fine‐tuned across clinical language, imaging and genomics, which learns representations that generalize across domains and produce high‐quality clinical reports, connecting genetic variation, imaging phenotypes and outcomes within a unified reasoning framework.

#### 3.3.2 Knowledge Augmentation and Innovation

Multimodal data opens new avenues for clinical reasoning by enabling AI systems to form richer, context‐aware representations of medical knowledge (Figure 5b). Public datasets that pair images with expert language are particularly catalytic. OpenPath couples pathology slides with natural‐language descriptions, facilitating vision–language pretraining tailored to diagnostic tasks [133], while LLaVA‐Med leverages large‐scale biomedical image–caption corpora from PubMed Central to acquire specialized domain knowledge and improve report generation and retrieval [134, 135]. Pretraining pathology‐focused vision‐language models on such resources has been shown to enhance diagnostic accuracy, promote knowledge sharing, and support case retrieval via either image or text prompts.

Beyond data scale, effective augmentation requires structured and interpretable knowledge. KGs provide a semantic scaffold that links entities and relations across molecular, cellular and systems levels, supporting compositional reasoning in precision medicine [136]. Scalable precision medicine oriented knowledge engine (SPOKE), a large‐scale biomedical KG from Bakar Computational Health Sciences Institute (BCHSI), encodes hierarchical relationships from pathways to phenotypes, enabling chain‐of‐thought–style inference and improving retrieval‐augmented generation (RAG) for medical LLMs [137]. Relying on EHRs alone is insufficient: therapeutic reasoning must integrate external, up‐to‐date domain knowledge from peer‐reviewed literature, textbooks and public ontologies [138]. Dynamic ingestion pipelines that continuously ground models in clinical trials and recent publications help maintain accuracy, scalability and generalizability as standards evolve [139, 140]. For example, literature‐mining frameworks can extract key signals (titles, keywords, abstracts, conclusions) across decades of PubMed to build diagnostically useful knowledge bases [141], while KnowLife aggregates insights from scientific articles, health portals and online communities to create a living repository for knowledge‐driven reasoning [142]. These pipelines lay the groundwork for cross‐domain learning by linking concepts across specialties and data types.

Crucially, multidomain AI is not only a conduct for codifying existing knowledge, but also a tool for discovery. Knowledge‐fusion approaches that align graphs with contemporary literature can reveal novel associations and treatment opportunities. For instance, graph‐augmented mining of recent publications has been used to prioritize drug repurposing candidates in Parkinson's disease, thereby accelerating hypothesis generation beyond traditional pipelines [81, 143].

## 4 Decentralizing Healthcare and Health Management

As populations grow, conventional care pathways struggle to scale: limited clinical workforce capacity is outpaced by demand [144, 145], and individual variability in physiology necessitates tailored decisions that strain provider time. These pressures are accelerating a shift toward computer‐augmented practice [146], spanning AI systems that flag imaging abnormalities and internet of things (IoT) tools that support telehealth, virtual visits and continuous patient–clinician communication [147, 148, 149].

**FIGURE 6:**
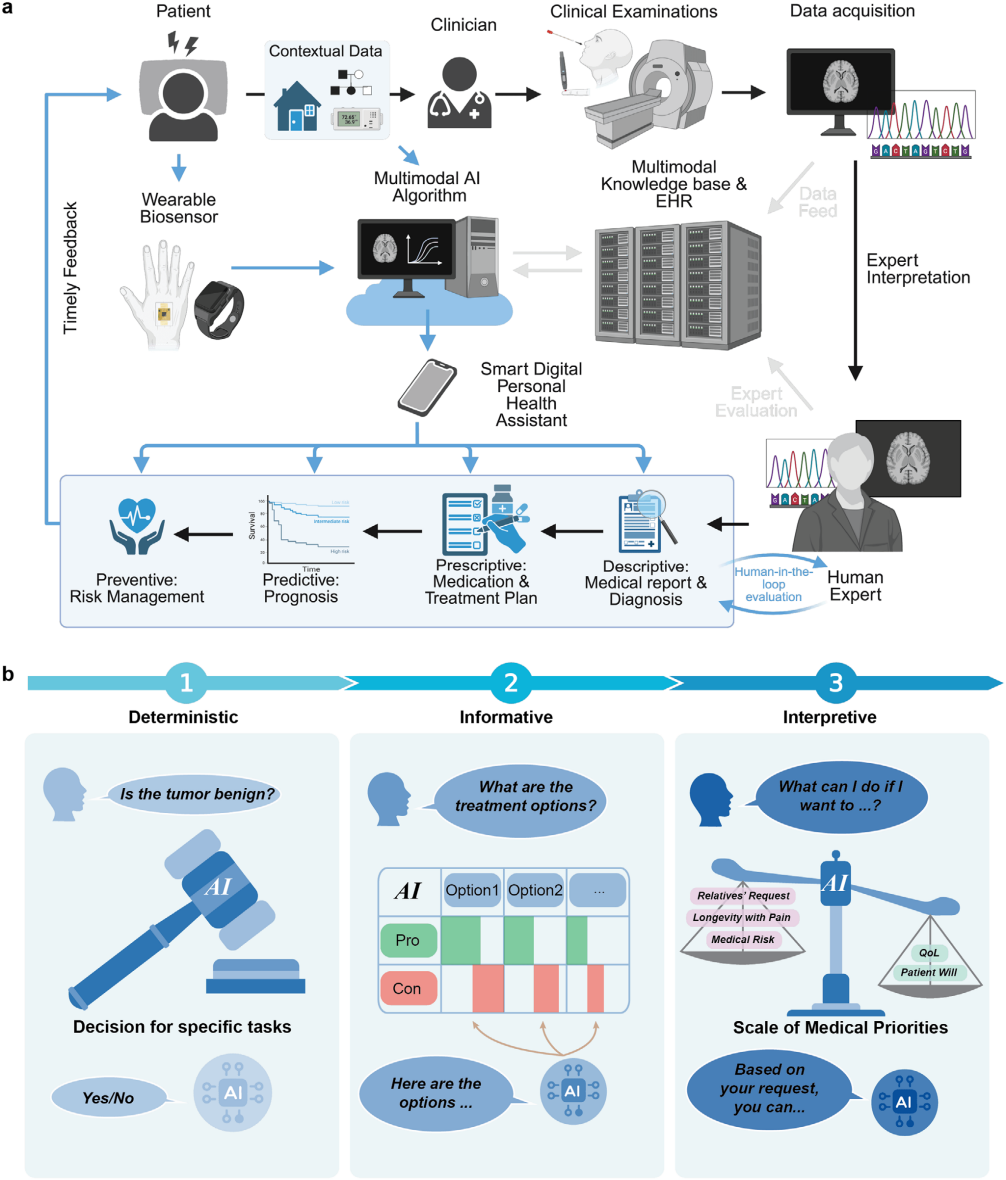
Democratizing healthcare with wearables and multidomain AI. Traditional healthcare pipeline (black arrows). (a) Blue arrows: an AI‐enabled pipeline where contextual data and wearable biosensors feed a multimodal AI system linked to EHRs/knowledge bases, returning guidance to a smart personal health assistant and clinician across descriptive reports, prescriptive therapy planning, predictive prognosis, and preventive risk management. Black arrows: the conventional workflow. Gray arrows: interactions between the traditional and AI‐augmented paths. (b) Outlook for patient‐specific assistants: a progression from deterministic (yes/no task decisions) to informative (option ranking with pros/cons) to interpretive systems that incorporate patient values, quality‐of‐life trade‐offs, and risk to recommend actions.

Integrating wearable biosensors with multidomain AI enables a patient‐centric model of care [150]. In the traditional pipeline (Figure 6a, black arrows), patients travel to clinics, undergo episodic testing, and await expert interpretation, an access barrier for those with geographic, mobility or socioeconomic constraints, and a workflow that captures only time‐point snapshots of chronic disease. By contrast, a biosensor‐plus‐AI pipeline (Figure 6a, blue arrows) continuously acquires real‐time physiology and streams it to a cloud‐based multidomain AI service. Fusing sensor signals with contextual data, the system returns expert‐like feedback, descriptive status reports, prescriptive recommendations, predictive risk forecasts and preventative guidance, delivered directly to patient devices for immediate support. Crucially, this paradigm supplements rather than replaces conventional care: as Meskó notes, AI should act as a critical adjunct to clinicians, not an alternative [151]. The democratized ecosystem remains coupled with traditional medicine (Figure 6a, grey arrows), expanding its knowledge base with EHRs and clinician expertise, which in turn improves the reliability and actionability of AI inferences over time.

### 4.1 Synergy of Biosensors and Multidomain AI

Marrying multidomain AI with wearable biosensors can shift care toward truly personalized, decentralized health management [152, 153]. Biosensors supply diverse, first‐hand physiological and behavioral streams, acquired continuously and in real time, data essential for dynamic models that adapt on the fly [3, 154]. In turn, multidomain AI elevates these devices from signal collectors to interpretive systems: through cross‐sensor fusion and integration, AI assembles a holistic view of health and captures interactions among physiological and environmental factors, delivering context‐aware analyses that account for activity, circadian timing, and other covariates [32]. This framework remains human centered: clinicians and domain experts are embedded in the loop to curate and audit the underlying knowledge bases, validate AI generated outputs as clinical suggestions rather than decisions, and guide iterative model refinement. This human oversight supports reliability, accountability, and clinical relevance while enabling the system to progressively learn individual baselines, facilitating earlier risk detection and proactive, preventive care. The result is tailored recommendations that refine over time as the system learns individual baselines and trends, enabling earlier detection of deterioration and proactive, preventive interventions.

Advances in wearables and IoT now extend telemedicine beyond the clinic, enabling continuous monitoring and remote diagnostics [39, 155]. Layering language model–powered agents onto this infrastructure is a natural next step: conversational interfaces can translate multimodal signals into medical‐grade summaries, personalized guidance and real‐time emotional support, closing the loop between sensing and action. Emerging “sense‐and‐respond” wearables further couple diagnostics with therapy, for example, automated drug delivery triggered by sensor thresholds, opening the door to responsive, autonomous health management. Together, these elements point toward smart biosensors: intelligent, adaptive interfaces that fuse rich biosensing with multidomain AI to deliver timely insight and intervention at the point of living.

#### 4.1.1 Improving Healthcare Delivery

**FIGURE 7:**
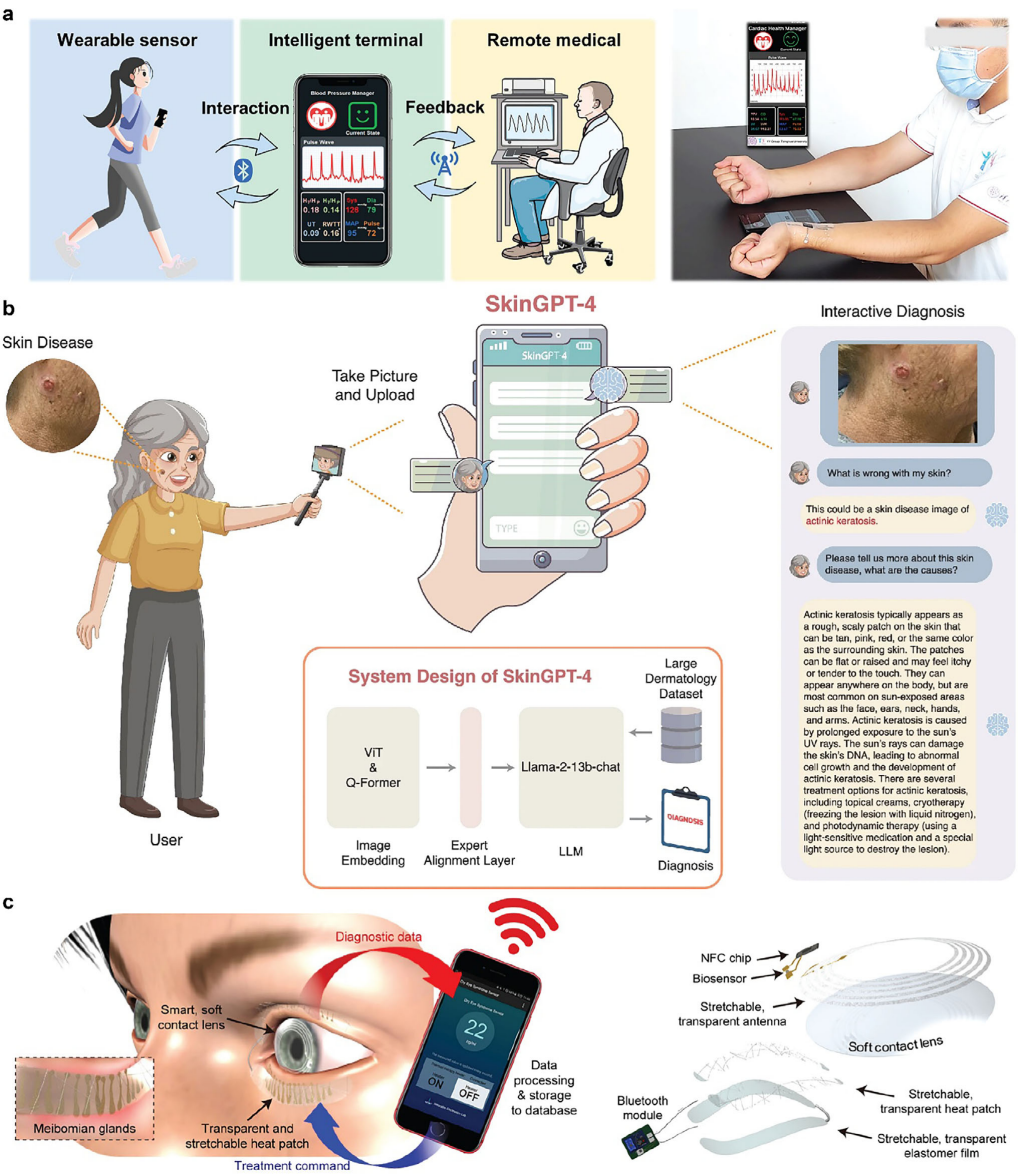
Case studies of decentralized healthcare. (a) Epidermal biosensor–enabled telemedicine with AI‐assisted self‐monitoring. Reproduced with permission [255]. Copyright 2023, The American Association for the Advancement of Science. (b) A GPT‐powered dermatology assistant that provides image‐based guidance and explanations. Reproduced with permission [69]. Copyright 2024, Springer Nature. (c) Mobile‐controlled theranostic platform combining a smart contact‐lens biosensor with a stretchable eyelid heating patch for ocular inflammation. Reproduced with permission [256]. Copyright 2021, The American Association for the Advancement of Science.

The convergence of advanced biosensors and multidomain AI can materially improve care delivery, advancing a more responsive and personalized system [156]. Health AI has progressed from deterministic systems that deliver binary or categorical outputs for narrowly defined clinical tasks, such as diagnosis or risk estimation, toward more nuanced forms of decision support. Deterministic AI offers consistency and efficiency, but it often frames medical decision making as an isolated, objective optimization problem, leaving limited room for clinical context or individual values. As model capability expands, AI can shift toward an informative role, moving from issuing decisions to presenting structured options. In this mode, AI compares alternatives, articulates benefits and risks, and makes tradeoffs explicit, informing clinicians and patients without dictating outcomes. The most human centered stage is interpretive AI, which situates medical options within a patient's broader priorities, including quality of life, pain tolerance, longevity, and personal values. Rather than optimizing a single clinical metric, interpretive AI helps balance competing considerations and supports shared, value aligned decision making (Figure 6b). Continuous, out‐of‐clinic monitoring of biomarkers via wearables offers a lifeline for underserved or mobility‐limited populations, enabling safe, at‐home tracking and clinician oversight even where infrastructure is sparse (Figure 7a). Through IoT connectivity, providers can review streaming data, issue real‐time feedback, and intervene promptly when thresholds are crossed.

Multidomain AI amplifies these benefits by functioning as a virtual clinician. By automating routine assessments and triage, AI reduces clinician workload and burnout, particularly during patient surges, freeing experts to focus on complex cases that demand judgement and empathy. [81, 157, 158]. For example, Xia et al. [159] introduced “Lingyi,” a multimodal KG–based medical conversational question answer (QA) system trained on large‐scale Chinese CQA data that delivers human‐like guidance via chat. Coupled with wearables, such agents can perform first‐line evaluation, alerting users to arrhythmias or dysglycaemia, recommending next steps, and escalating to human care when warranted.

By decentralizing access, these tools empower individuals to manage health proactively and receive the proper care at the right time, irrespective of geography or physical constraints, yielding a more resilient, responsive and inclusive healthcare ecosystem. A related shift is the integration of mobile diagnostics with digital clinical trials, which pushes research and care closer to everyday life [160, 161]. Portable sensors and smartphone platforms support continuous, real‐world data capture; paired with remote trial participation, they broaden inclusion, accelerate insights and enable personalized interventions. Realizing this vision requires rigorous data‐quality assurance, robust multimodal integration, and alignment with evolving regulatory frameworks. As adoption grows, multimodal AI that unifies diverse streams, physiological, behavioral and contextual, will become essential for delivering accessible, personalized and equitable healthcare on a large scale.

#### 4.1.2 More Personalized Healthcare

Physiology varies widely across individuals, shaped by genetics, lifestyle, environment and medical history, so population “normals” may be inappropriate for any given person. Continuous streams from wearable biosensors move beyond episodic snapshots to capture each individual's dynamic baseline. A modest rise in blood pressure, for example, may be clinically meaningful for someone with a low baseline, even if values remain within population ranges. Such longitudinal, person‐specific data enable proactive care tuned to individual trajectories rather than cohort averages. Efforts to couple clinical indicators with biosensor outputs are building the infrastructure for this shift. The AI4Food database, for instance, aggregates biosensor measurements alongside multiple physiological markers to link nutrient status, behavior and health, supporting finer‐grained personalization [162].

Multidomain AI amplifies these gains by integrating continuous biosensor signals with contextual and empirical information (e.g., activity, stress, diet, medications). These fusion yields recommendations that are both evidence‐based and tailored, optimizing therapies, reducing side effects and improving adherence. Deployed through conversational agents, these models can guide users through their data, explain anomalous readings and suggest next steps in real time (Figure 7b), enabling people to actively participate in decisions and to manage their health with greater confidence.

#### 4.1.3 In Situ Treatment

The marriage of wearable biosensors with multidomain AI enables on‐site (in situ) therapy, where sensing, inference and actuation occur within a single, body‐conformal system (Figure 7c). This is pivotal for time‐critical events, stroke, seizures, severe dysglycaemia, when seconds determine outcomes and clinic‐based care is unreachable. Continuous monitoring establishes individualized baselines; AI identifies clinically meaningful deviations, quantifies risk, and initiates precisely titrated, context‐aware interventions [163].

Seizure care illustrates the approach. Joo et al. [164] developed a soft, implantable drug (SID) platform that monitors EEG and automatically releases subcutaneous therapeutics at onset, mitigating episode severity and improving recovery prospects. In diabetes management, Zhang et al. [165] reported a glucose‐sensing e‐skin that closes the loop by titrating insulin in real time, stabilizing glycaemia and reducing hypoglycemic and hyperglycemic excursions beyond what manual regimens can achieve. Oral health offers a further example: Shi et al. [166] introduced a wearable dental patch that tracks electrochemical status and delivers fluoride on demand; AI assessment of the local environment anticipates demineralization risk and times dosing to maximize protection while minimizing exposure.

Importantly, real‐world deployment hinges on rigorous safeguards, calibration drift detection, multi‐signal confirmation to suppress false positives, dose caps, clinician override and audit trails, integrated with secure connectivity for remote supervision. With these controls, sensor‐guided, AI‐orchestrated actuation transforms passive monitoring into responsive, personalized therapy at the point of living.

#### 4.1.4 Interpretive Medical Assistant

AI‐enabled medical assistants have progressed through three phases, deterministic, informative and interpretive, driven by advances in learning architecture and multimodal reasoning. From the perception onward, models were specialized, task‐bound and rule‐ or feature‐driven. Even with the advent of deep learning in 2006, systems largely produced fixed mappings from inputs to outputs, supporting narrow applications such as image‐based anomaly detection or risk scoring from structured records, useful, but context‐poor and non‐interactive [167, 168]. Transformer‐based language models (GPT, GPT‐2, GPT‐3; 2018–2020) enabled fluent, context‐aware dialogue [169]. Agents could retrieve and synthesize medical knowledge, explain differential diagnoses and outline treatment options based on patient history, evolving from single‐task tools to knowledgeable assistants capable of richer, consultative exchanges.

With GPT‐4 class multimodal models, assistants begin to reason across modalities (text, images and time series), adapt to patient goals and participate in shared decision‐making. Rather than delivering static answers, agents incorporate feedback, reconcile conflicting signals and update recommendations as circumstances change. Early studies illustrate this shift: Li et al. [170] report an image‐grounded LLM (DeepDR‐Transformer/DeepDR‐LLM) that supports diabetes management and diabetic‐retinopathy referral, matching endocrinology trainees in English and surpassing primary‐care performance in Chinese, while improving self‐management and the empathy of recommendations. Wan et al. [171] develop a site‐specific prompt‐engineering chatbot (SSPEC) trained on >35k real outpatient–nurse dialogues, achieving higher task‐completion with fewer turns and enabling a nurse–agent collaboration workflow for uncertainty handling. In essence, the trajectory is from predefined mappings to contextual explanation and now to adaptive, multimodal interpretation. As interpretive assistants integrate domain knowledge, patient preferences and streaming biosensor data, they are poised to become collaborative partners, bridging rigid computation and clinician judgment to enhance care delivery and patient outcomes.

### 4.2 Future Challenges

#### 4.2.1 Data Privacy

Multimodal health records, diagnoses, images, streaming biosensor traces, and genomics encode highly personal information, and sharing them safely requires end to end safeguards spanning identity, storage, transmission, and access control [172, 173]. Protecting patient data is foundational to cross domain integration, and compliance with established regulatory frameworks such as the EU's General Data Protection Regulation (GDPR) and the United States’ Health Insurance Portability and Accountability Act (HIPAA) defines the minimum acceptable standard for privacy protection. These frameworks shape permissible data flows, impose governance and accountability requirements, and ensure that multimodal biosensing systems retain patient agency and legal compliance.

To meet these regulatory obligations, privacy‐preserving data transformation and de‐identification, consistent with the HIPAA Safe Harbor method and the GDPR Recital 26 standard for anonymization, have become essential at the data level. Cryptographic and encryption methods can convert medical data into protected representations that are difficult to reidentify [174, 175]. Neural network‐based image encryption schemes can further map medical images into encrypted latent representations while preserving clinically relevant content [176, 177]. Distortion based anonymization suppresses identity bearing structures while retaining task relevant signal for downstream learning, and noise injection can help obscure latent data structure [178]. Synthetic data generation and privacy preserving embeddings via generative models, such as variational autoencoders (VAEs) and generative adversarial networks (GANs), can further reduce exposure by enabling institutions to share semantically useful representations rather than raw data [179]. These point level protections integrate with federated learning and secure multi‐party computation, which keep data local while transmitting only encrypted gradients or aggregated updates.

At the systems level, secure architectures reinforce these privacy preserving techniques. As Internet of Medical Things (IoMT) deployments expand, conventional authentication schemes face risks including privacy leakage, opaque malicious activity, and single points of failure during cross institutional exchange. Blockchain based designs offer decentralization, auditability, and policy automation via smart contracts [173], with practical hardening through double anonymity and three factor authentication. Decentralized designs can also mitigate insider threats in wireless medical sensor networks by distributing patient data across multiple servers. By ensuring that no single administrator can access the full dataset and enabling computation without decryption, such approaches protect privacy even against insiders with database privileges [180]. These techniques can reduce overhead without sacrificing security, supporting trustworthy multimodal sharing for AI assisted reasoning [181]. In addition, adherence to structured metadata standards such as fast healthcare interoperability resources (FHIR) supports consistent representation of patient information, temporal context, and derived features across institutions. Complementarily, traceability‐enhancing methods can embed identity‐linked digital watermarks so that copies of records remain attributable, enabling accountability if data are modified, misused, or leaked [182]. Standardized metadata frameworks and provenance management mechanisms support safe adjudication by providing auditable, interoperable, and verifiable clinical data flows.

However, technical safeguards alone are insufficient without operational transparency, which determines how data are governed, accessed, and communicated to patients and clinicians [183]. Users must retain meaningful control over their personal data [184] and understand the data pathway, including what transformations are applied, where data are stored, who can access specific segments, and under what justification [185]. Under the GDPR data minimization principle, operational policies should constrain data collection to what is clinically necessary in scope, modality, resolution, and duration, with defaults favoring minimal acquisition rather than maximal observability. This is particularly important in human in the loop settings, such as clinician review of AI generated alerts, manual override of recommendations, and audit log inspection. Consistent with GDPR Recital 32, dynamic consent mechanisms implemented through tangible electronic interfaces, such as web‐based patient dashboards [186, 187] or mobile consent applications [188], can allow patients to adjust permissions or withdraw participation as system use evolves [189]. Beyond generic opt‐in or opt‐out models, these interfaces should also support concrete consent limits, including time‐bounded collection, such as excluding nighttime monitoring, modality‐specific permissions, geolocation restrictions, and symptom‐triggered activation of sensing. To further reduce downstream disclosure risks, privacy protections should extend to population‐level summaries through differential privacy mechanisms [190, 191], reducing the likelihood that aggregate analytics can be reverse‐engineered to infer individual participation. An explicit end‐to‐end workflow should ensure that consent is captured through the interface, followed by data transformation or anonymization consistent with the granted permissions, and that all subsequent access events are logged, auditable, and subject to review. Accordingly, systems should make transparent: (*i*) data processing steps; (*ii*) the parties authorized to view or modify each data type; and (iii) residual risks, such as re identification probability or unauthorized access despite safeguards. Dynamic consent mechanisms are also needed to allow patients to adjust permissions or withdraw participation as system use evolves. Ultimately, strong encryption, granular access control, and verifiable anonymization pipelines are core infrastructure for decentralized, AI enabled healthcare. Robust privacy engineering across data, system, and operational layers is a prerequisite for safe and scalable use of multimodal health data.

#### 4.2.2 Trustworthiness and Public Acceptability

Translating the synergistic biosensing and multidomain AI framework into a practical decentralized healthcare system that is acceptable to the public requires stepwise, multifaceted validation demonstrating safety, usability, and non‐inferiority to standard care.

Evaluating AI generated decisions requires more than assessing raw signal accuracy. It also requires evidence that recommendations follow coherent reasoning and align with clinically acceptable choices. Decision validity can first be assessed through comparison with case specific gold standard benchmarks or expert annotated datasets, with concordance quantified using metrics such as exact and weighted agreement, precision and recall, and expert panel appropriateness scores [192]. Accordingly, expert constructed, scenario‐based benchmark datasets are essential for evaluating response quality across clinically relevant conditions [193, 194]. Beyond static datasets, counterfactual testing can expose the model to diverse clinical vignettes, including edge case or high‐risk situations, to verify that recommendations remain safe, consistent, and clinically plausible across varying biomarker patterns, comorbidity profiles, and temporal states. For example, decision support from AI can be evaluated using specific clinical vignettes, such as a 65‐year‐old patient presenting with sudden chest pain, elevated troponin, and a history of diabetes and hypertension. This layered evaluation helps ensure that the decision support module captures clinically meaningful structure rather than relying on superficial correlations, and that its outputs remain robust under the real‐world variability anticipated in deployment.

Because decision support systems ultimately operate within users in the loop workflows, their safety and effectiveness depend not only on recommendation quality but also on how users, including both patients and clinicians, interpret, trust, and act on model outputs. Human AI interaction quality should therefore be evaluated through structured human in the loop studies [195] in which users manage simulated or prospective cases with and without AI assistance [196]. Interpretability is equally important [197], including whether explanations, and uncertainty estimates are consistent with clinical reasoning and enable transparent justification of recommendations. Temporal alignment and causal reasoning are critical to ensure that AI recommendations reflect clinically meaningful dependencies. For example, tracking how biomarker levels evolve over time in patients with comorbidities can help models infer intervention priorities based on plausible temporal relationships rather than static correlations. Such evaluations can be supported by interpretability and causal analysis tools, including feature attribution [198], attention maps [199], causal graphs [200, 201], and counterfactual explanations [197], which probe decision sensitivity and help distinguish clinically meaningful dependencies from spurious associations [202]. In parallel, trustworthy deployment requires explicit assessment of model calibration, ensuring that predicted risks and confidence estimates correspond to observed outcomes [203]. Calibration can be quantified using established metrics such as expected calibration error [204], the Brier score [205], and reliability diagrams [206], which are particularly important in safety‐critical settings where overconfidence may lead to inappropriate clinical action. A comprehensive, practice‐oriented overview of calibration metrics is provided by Cabitza et al. [205]. Because wearable‐AI systems operate longitudinally, interpretability and calibration should be complemented by continuous drift monitoring, including tracking changes in input distributions, embedding spaces, and performance metrics over time [207].

A further challenge is public acceptability. Patients may still prefer human clinicians over AI advice, and acceptance may decline when algorithms influence treatment selection [208, 209, 210, 211]. Core concerns include opacity, or black box reasoning, and hallucination, in which outputs appear plausible yet lack sufficient evidentiary support and may misdirect diagnosis or therapy [212]. Mitigations therefore require model governance, including prospective validation, distribution shift monitoring, and defined fallback pathways, together with transparent inference through faithful rationales and uncertainty quantification, and grounded generation. Retrieval augmented generation can improve fidelity by tethering responses to verifiable sources, strengthening both accuracy and traceability. Domain adaptations such as MedGraphRAG provide evidence linked answers on private medical data, while multimodal RAG that fuses clinical notes, time series EHR, and external knowledge graphs, for example PrimeKG, has shown improved predictive performance by injecting context that may be missing from pretraining.

However, because clinical knowledge evolves continuously, trustworthy use of knowledge graphs also requires explicit provenance tracking and version control [213]. Recommendations should be traceable to the specific knowledge‐graph snapshot, source ontology, and update timestamp used at inference time, enabling post hoc auditing, reproducibility, and clinical accountability [214]. Versioned knowledge graphs, together with changelogs documenting added, deprecated, or revised relations, allow systems to distinguish outdated from current evidence and to flag recommendations that may be affected by subsequent knowledge updates. At early stages of AI assisted decision making, human in the loop review remains necessary to ensure decision quality and to detect unsafe or misleading outputs. Ultimately, trustworthy deployment pairs technical safeguards with human oversight, clear accountability, and communication that respects patient values.

#### 4.2.3 Scalability and Generalizability

Although multimodal models consistently outperform unimodal baselines, scaling and generalizing them for real world clinical deployment remains challenging. Barriers to generalizability in multimodal biosensing systems are inherently multidimensional and span three interdependent axes, population, device, and use case generalization [215]. The population axis is constrained primarily by dataset imbalance, where specific demographics, disease phenotypes, or care pathways are often overrepresented [215]. Such imbalance, together with physiological variability across age groups, individual differences, and evolving disease trajectories, can undermine reliability when models are applied to underrepresented populations [216, 217]. The device axis is challenged by heterogeneous sensing hardware that introduces structured noise, inconsistent sampling rates, and sensor drift [218]. These issues are further compounded by difficulties in spatial and temporal registration when integrating asynchronous multimodal streams, which can weaken alignment or mask key features. The use case axis reflects variation in care environments, including hospital versus home monitoring, differences in clinical workflows, and uneven availability of sensing modalities [219]. In real world settings, incomplete or missing modalities are common due to resource constraints, patient adherence, or device failure, requiring systems to operate robustly under partial observability [220, 221]. Collectively, data noise, missing modalities, registration challenges, and dataset bias represent core barriers to scalable and deployable multimodal healthcare applications [222]. Addressing these systemic challenges therefore requires a layered set of strategies.

First, at the data representation level, effective generalization depends on reliable alignment across heterogeneous modalities, because discrepancies in temporal resolution, sampling rates, and sensor dynamics can propagate downstream and degrade learned representations. Standardized multimodal benchmark datasets, such as those curated by PhysioNet [223], including MIMIC waveform and challenge datasets that combine high‐resolution physiological time series, laboratory measurements, and clinical outcomes, and ADNI [224], which integrates longitudinal neuroimaging, cognitive assessments, and molecular biomarkers for neurodegenerative disease, provide synchronized or partially aligned recordings across modalities. These resources can be used to evaluate alignment quality, fusion strategies, and generalization across populations and sensing platforms. Complementarily, structured biomedical knowledge resources such as SPOKE [137] enable benchmarking of multimodal reasoning and cross‐domain knowledge integration, rather than raw signal alignment. Alignment quality can be quantified using established metrics such as dynamic time warping [225] and cross‐correlation [226] to assess temporal synchrony, as well as reconstruction error [227] to evaluate whether cross‐modal mappings preserve clinically meaningful signal structure. Based on reliable aligned data, embeddings that capture clinically meaningful structure can be learned while minimizing sensitivity to modality specific artifacts. Self‐supervised and contrastive objectives across heterogeneous cohorts encourage models to focus on shared structural and temporal dynamics rather than population or device specific noise patterns [228]. Incorporating modality dropout and partial input training further prepares models to operate under incomplete or intermittently missing modalities, which is common in resource limited settings [229]. Generative and interpolation models may also help address data imbalance by synthesizing plausible samples or estimating missing segments to improve coverage of underrepresented conditions [230, 231].

Second, at the model training level, robust generalization across populations, devices, and clinical settings requires learning paradigms that can accommodate evolving data. Continual learning provides a mechanism for models to incrementally incorporate new cohorts, devices, and modalities while preserving prior knowledge [232]. This capability is particularly important in clinical contexts where patient popdeginedulations shift, hardware platforms are updated, domain knowledge expands, and deployments extend across multiple stages of care. By retaining previously acquired representations while adapting to new distributions, continual learning can expand applicability without repeated retraining from scratch. Complementarily, federated learning can support generalization by exposing models to heterogeneous institutional environments without centralized data aggregation [233]. Because participating institutions contribute data that differ in modality completeness and synchronization quality, federated learning can implicitly promote robustness to missing modalities and imperfect spatial and temporal registration [234].

In addition to training paradigms, rigorous evaluation strategies are essential for validating generalizability prior to deployment. Stratified subgroup evaluation assesses model performance across predefined population, device, and use‐case strata, rather than relying on aggregate metrics that may obscure failure modes [235, 236]. For population‐level generalization, models should be evaluated across demographic and clinical subgroups defined by age, sex, disease phenotype, comorbidity profiles, or care pathways [237]. This helps safeguard under‐represented populations and demonstrates that learned representations generalize beyond the dominant training distribution, supporting safer translation to real‐world clinical environments. Because deployments are inherently longitudinal, post‐deployment surveillance is also critical [238]. Models should undergo periodic bias audits to monitor shifts in subgroup performance as data distributions, clinical practices, and patient populations evolve.

Performance metrics such as error rate, discrimination, and calibration should be assessed not only in aggregate [239] but also under explicit robustness protocols [240], including cross‐site validation, leave‐one‐site or leave‐one‐device‐out evaluation, and device‐shift stress tests that reflect changes in sensing hardware or care environments [241]. Degradation under these conditions, quantified by AUROC drop and changes in calibration error relative to in‐distribution baselines, provides a practical measure of generalizability and aligns evaluation with regulatory expectations for robustness and post‐market performance monitoring in clinical AI systems throughout the model lifecycle [242].

#### 4.2.4 Regulatory Co‐Governance

When considering deployment of multimodal wearable–AI systems, it is essential to adhere to governance frameworks that span both physical sensing devices and adaptive software components. Existing regulatory pathways define rigorous pre‐market evaluation for sensing hardware and software functions. In the United States, AI and analytics functions can be regulated as Software as a Medical Device (SaMD) [243, 244], while physical biosensing hardware remains subject to conventional premarket pathways. In the European Union, integrated sensing–AI systems are governed under the Medical Device Regulation (MDR) [245], with risk considerations informed by International Medical Device Regulators Forum (IMDRF) frameworks that account for intended use, autonomy, and potential clinical impact [246]. Quality management systems such as ISO 13485 can be applied to impose design controls, risk management, and post‐market surveillance obligations across both hardware and software [247]. Furthermore, because decentralized wearable platforms often rely on cloud infrastructure and cross‐institutional data exchange, international deployment must address cross‐border data governance [248], including GDPR transfer mechanisms for data movement outside the European Union and HIPAA Business Associate Agreements (BAAs) for protected health information in the United States.

Despite the existence of formal regulatory pathways for medical software, legal and ethical frameworks remain less mature for adaptive and data‐driven AI systems, particularly as models increase in complexity and autonomy [249]. Many regulatory mechanisms were developed for static algorithms whose behavior remains fixed after approval, an assumption increasingly misaligned with modern machine learning paradigms. For example, Food and Drug Administration (FDA) authorization of the autonomous diabetic retinopathy system, IDx‐DR, required the algorithm to be locked at the time of approval, limiting post‐deployment adaptation and continual learning [250]. This framing treats the AI as a non‐learning diagnostic device, constraining pathways for controlled improvement over time.

These challenges are further amplified by the emergence of large language models and foundation models in medicine, which are often pretrained on heterogeneous data, updated iteratively, and repurposed across tasks and clinical contexts [251]. Such models blur traditional boundaries between development, validation, and deployment, raising unresolved questions about accountability, version control, distribution shift, and post‐market performance monitoring [252]. As a result, current regulatory approaches can struggle to balance innovation with safety when performance and behavior may evolve as new data and updates are introduced [253].

These limitations motivate regulatory frameworks that extend beyond point‐in‐time approval toward lifecycle‐based governance of integrated sensor–AI systems. Multimodal wearable platforms combine sensing hardware, data pipelines, cloud infrastructure, and adaptive analytics into tightly coupled systems whose clinical risk emerges from their interaction rather than from any single component. Regulating hardware and software in isolation is therefore insufficient to capture real‐world safety, reliability, and clinical impact. Future regulatory models will need to incorporate continuous performance evaluation, controlled updating, real‐world evidence generation, and traceable change management across both hardware and software. Based on existing institutions and standards, including FDA, IMDRF, and MDR mechanisms, such lifecycle‐based governance can better address adaptive behavior, post‐deployment updating, and cross‐border data dependencies within a unified oversight structure. This alignment with the realities of modern multimodal systems is essential to enable responsible innovation while preserving patient safety, trust, and clinical accountability across the operational lifecycle of wearable–AI technologies.

### 4.3 Roadmap for Real‐Life Deployment

A pragmatic near term starting point is to anchor multimodal sensing and multidomain AI within a single, well bounded clinical scenario where disease mechanisms are established and risk is comparatively low. Chronic conditions such as diabetes represent a particularly realistic starting point because: *(* *i* *)* pathophysiology and treatment algorithms are mature, providing a well‐developed knowledge base for decision support; *(* *ii* *)* disease progression is relatively slow, creating a lower risk environment with a larger window for intervention; *(* *iii* *)* key biomarkers are accessible to wearables, including blood and interstitial fluid glucose dynamics, and are routinely interpreted in clinical practice; *(* *iv* *)* commercial wearables already provide reliable monitoring of core biomarkers, for example continuous glucose monitoring platforms such as Dexcom, which can serve as a practical entry point for multimodal integration; and *(* *v* *)* reimbursement pathways are comparatively mature, including established Current Procedural Terminology (CPT) coding for continuous glucose monitoring, offering a precedent from which broader monitoring and decision support models may evolve. These properties make diabetes an effective proving ground for decentralized healthcare models (206). Scalability in decentralized healthcare will depend less on proliferating condition specific devices than on reusing modular multimodal architectures across clinical verticals. Case studies such as wound care and ocular monitoring therefore serve as exemplary of transferable design patterns rather than endpoints of application.

From a human‐centered design perspective, multimodal wearable–AI systems should be embedded within a minimal, auditable workflow that preserves clinician authority and patient agency. In an ideal deployment scenario, patients provide dynamic consent through a dedicated interface, after which wearable data are collected and transformed in accordance with the approved permissions. AI models generate risk estimates or decision‐support outputs that are presented to clinicians alongside uncertainty and rationale, enabling review, contextual interpretation, and override when appropriate. Clinical actions remain the responsibility of human decision makers, while all data access, model versions, and decision events are logged to support accountability and post hoc auditing. This workflow ensures that AI augments rather than replaces human judgment and aligns system operation with clinical practice.

At scale, multimodal wearable biosensor–AI systems are likely to be deployed through Remote Patient Monitoring (RPM) and Remote Therapeutic Monitoring (RTM) paradigms, which provide established clinical, operational, and reimbursement frameworks for decentralized care. These programs define how continuous sensing data are collected, reviewed by care teams, integrated into electronic health records, and translated into reimbursable clinical actions, making them a natural substrate for operationalizing multimodal biosensing and AI‐assisted decision support. In addition, RPM and RTM align with value‐based care incentives by shifting reimbursement from episodic encounters toward continuous risk management, earlier intervention, and outcome‐based monitoring, thereby creating financial pathways that can sustain deployment beyond pilot studies.

Looking ahead, we outline three milestones. Near term priorities focus on strengthening the technological foundation, including improved sensor robustness, standardized cross modality calibration and synchronization, benchmark dataset development, and interpretable, uncertainty aware AI pipelines. Mid‐term goals center on clinical translation, including multi‐site prospective trials, interoperable standards for integrating EHR and biosensor data, practical regulatory strategies, and reimbursement models that reward proactive, continuous monitoring. Longer term milestones require scalable manufacturing pipelines for both hardware and software, together with a mature ethical, legal, and policy governance ecosystem that supports equitable and trustworthy deployment. These phased targets define a realistic path from targeted, condition specific decision support tools to system level deployment of multimodal biosensing and multidomain AI in routine healthcare.

## 5 Conclusion

The integration of multimodal biosensing with multidomain AI charts a path from continuous physiological data streams to clinically meaningful decisions, reshaping care to be more accessible, personalized, and decentralized. Continuous, real‐time acquisition of diverse bioindicators, coupled with AI agents grounded in clinical domain knowledge and knowledge graphs, enables monitoring beyond the clinic and elevates systems from signal interpretation to higher order reasoning and tailored recommendations. Emerging large language models further add a conversational interface that translates complex outputs into actionable guidance, supports self‐management, and provides timely, empathetic feedback, helping reduce reliance on facility‐based care and enabling intervention earlier.

Realizing this vision will require stepwise advances in robust and trustworthy cross modality fusion, reliable wearable sensing hardware, interoperable standards for heterogeneous health data, and rigorous privacy and security safeguards. Key priorities include seamless integration of biosensor networks with knowledge driven AI at the edge, transparent benchmarking across populations and conditions, and human centered system design that preserves clinician oversight. With coordinated interdisciplinary effort, the partnership between multimodal biosensing and multidomain AI can give individuals greater control over their health, accelerate the transition from sensors to decisions, and advance a more equitable and proactive model of care.

## Conflicts of Interest

The authors declare no conflict of interest.

## Data Availability

The authors have nothing to report.
